# Soil proteogenomics uncover microbial strategies for soil depth-specific carbon cycling under whole-soil warming conditions

**DOI:** 10.1038/s41467-026-78137-0

**Published:** 2026-10-13

**Authors:** Ulas Karaoz, Ricardo J. E. Alves, Nikola Tolic, Rosalie K. Chu, Carrie Nicora, Jason Toyoda, Emiley Eloe-Fadrosh, Margaret S. Torn, Eoin L. Brodie

**Affiliations:** 1 Climate and Ecosystem Sciences Division, Lawrence Berkeley National Laboratory, Berkeley, CA USA; 2 Environmental Molecular Sciences Laboratory, Pacific Northwest National Laboratory, Richland, WA USA; 3 DOE Joint Genome Institute, Lawrence Berkeley National Laboratory, Berkeley, CA USA; 4 Energy and Resources Group, University of California, Berkeley, CA USA; 5 Department of Environmental Science Policy & Management, University of California, Berkeley, CA USA

**Keywords:** Soil microbiology, Carbon cycle, Data integration, Microbial communities

## Abstract

Soils are Earth’s largest carbon reservoir, yet microbial mechanisms controlling decomposition, and their warming response, remain poorly resolved, especially in subsoils. Using genome-resolved proteogenomics within a replicated whole-soil warming experiment (0–90 cm, +4 °C, 7.5 years) we map microbial traits, their depth stratification, and warming responses. Depth is the dominant driver of microbial community and trait structure. Depth-wise, metabolism converge on low-molecular-weight and C1 compounds, challenging glucose-centric models, with surface soils enriched in methanol oxidation and deep soils in alcohol fermentation, CO oxidation and N₂ fixation. Abundant sialidases in deep soils implicate glycoproteins as a key carbon source. Warming effects are smaller and decrease over time but are depth-specific: surface soils show enhanced amino sugar and sugar alcohol transport, phosphorus mobilization, and stress tolerance, whereas mid- and deep soils exhibit stimulated C1 metabolism, CO oxidation, and altered nitrogen turnover. These depth-specific microbial trait shifts may critically influence soil carbon–climate feedbacks.

## Abstract (web summary)

In this study, the authors use proteogenomics of a whole-soil warming experiment to show that soil depth dictates microbial community and trait structure more than soil warming. Deep-soil microbes rely on alternative survival strategies and actively produce enzymes that break down complex glycoproteins.

## Introduction

Microbial transformation of plant inputs to soil is the source of most soil organic matter (SOM), driving soil carbon and nutrient cycling^1–4^, which releases significantly more CO₂ to the atmosphere^5^ than all anthropogenic sources combined^6^. Although subsoils (>30 cm depth) contain over half of the global soil carbon stocks^7^, comparatively little is known about the microbial communities, substrates, and traits below the topsoil. Soil properties, nutrient inputs, and SOM chemistry shift markedly with depth^8–10^ due to the declining influence of roots and aboveground inputs, altered mineral–organic interactions, and long-term soil development processes^11–13^. These depth-dependent conditions are expected to select for microbial communities with distinct metabolic, physiological, and cellular properties, i.e., microbial traits. However, there is still limited understanding of decomposition processes and the cycling of carbon and nutrients in subsoils.

As a biochemical process, SOM decomposition is inherently temperature-dependent, yet the mechanisms by which microbial communities mediate decomposition under warming conditions remain poorly understood. Most ecosystem biogeochemistry models neither explicitly represent depth-dependent heterogeneity nor incorporate microbial traits^14^, even though microbes likely govern soil biogeochemical responses to warming across the depth profile. This lack of representation contributes to large uncertainties in climate–carbon cycle feedbacks^15,16^.

Microbial traits, such as metabolic potential, growth rate, and biomass stoichiometry, determine functional plasticity and activity rates. They regulate substrate uptake and allocation between biomass production, respiration (CO₂ release), and nutrient turnover. Trait distributions vary over space and time as microbes both respond to, and modify, their environments^17–20^. Depth gradients in plant inputs, substrate quality, nutrient availability, moisture, and temperature create distinct microbial habitats, selecting for divergent functional traits^21–25^. Consequently, reassembly of microbiomes and their traits is expected to vary with depth under warming, potentially leading to complex, phased responses across the soil profile^26^.

To address these gaps, we leveraged a long-term whole-soil warming experiment in a temperate conifer forest (Sierra Nevada, CA; Blodgett Forest Research Station), where the entire soil profile has been continuously warmed by 4 °C above ambient temperature since 2014. This treatment has increased annual soil respiration by 30–37%, including proportional responses from subsoils^27^. Previous work at this site showed that microbial responses to warming are not uniform across depths: communities in deeper soils change more slowly than those in surface soils^28^. We hypothesize that depth-stratified microbial traits, in combination with their environmental context, such as substrate energetics and stoichiometry, and mineral interactions, mediate the long-term response of soil respiration to warming.

Here, we apply deep metagenomic sequencing and metaproteomics (proteogenomics) to soils spanning 0–90 cm depth and up to 7.5 years of warming. Our goals are to determine how microbial traits, their distribution across genomes, and their expression vary with soil depth, and to evaluate how their selection and activity are altered by experimental warming. Specifically, we ask: (i) how do microbial communities and their traits stratify with depth, and what insights does this provide about why decomposition rates and carbon storage change with depth? (ii) How do microbial trait distribution and expression respond to long-term warming? and (iii) which substrates and metabolic pathways are central to these responses?

Across a 0–90 cm soil profile subjected to long-term whole-soil warming, microbial community composition and functional potential were strongly structured by depth, which explained substantially more variation than warming. Surface communities were enriched in methanol/formaldehyde metabolism and uptake of plant-derived substrates, whereas deep soils showed greater potential for CO oxidation, alcohol fermentation, nitrogen fixation, and hydrogen metabolism. Warming produced comparatively smaller, depth- and time-dependent shifts, enhancing carbohydrate and organic nutrient uptake and stress-tolerance traits mainly near the surface, while altering C1 and nitrogen-cycling functions in mid- and deeper soils. Proteomics confirmed in situ expression of these depth-specific pathways, including widespread C1 metabolism and unexpectedly abundant sialidases and carbohydrate-active enzymes in deep soils.

## Results

### Extensive genomic sampling of microbial members from deep soil profiles

**Fig. 1:**
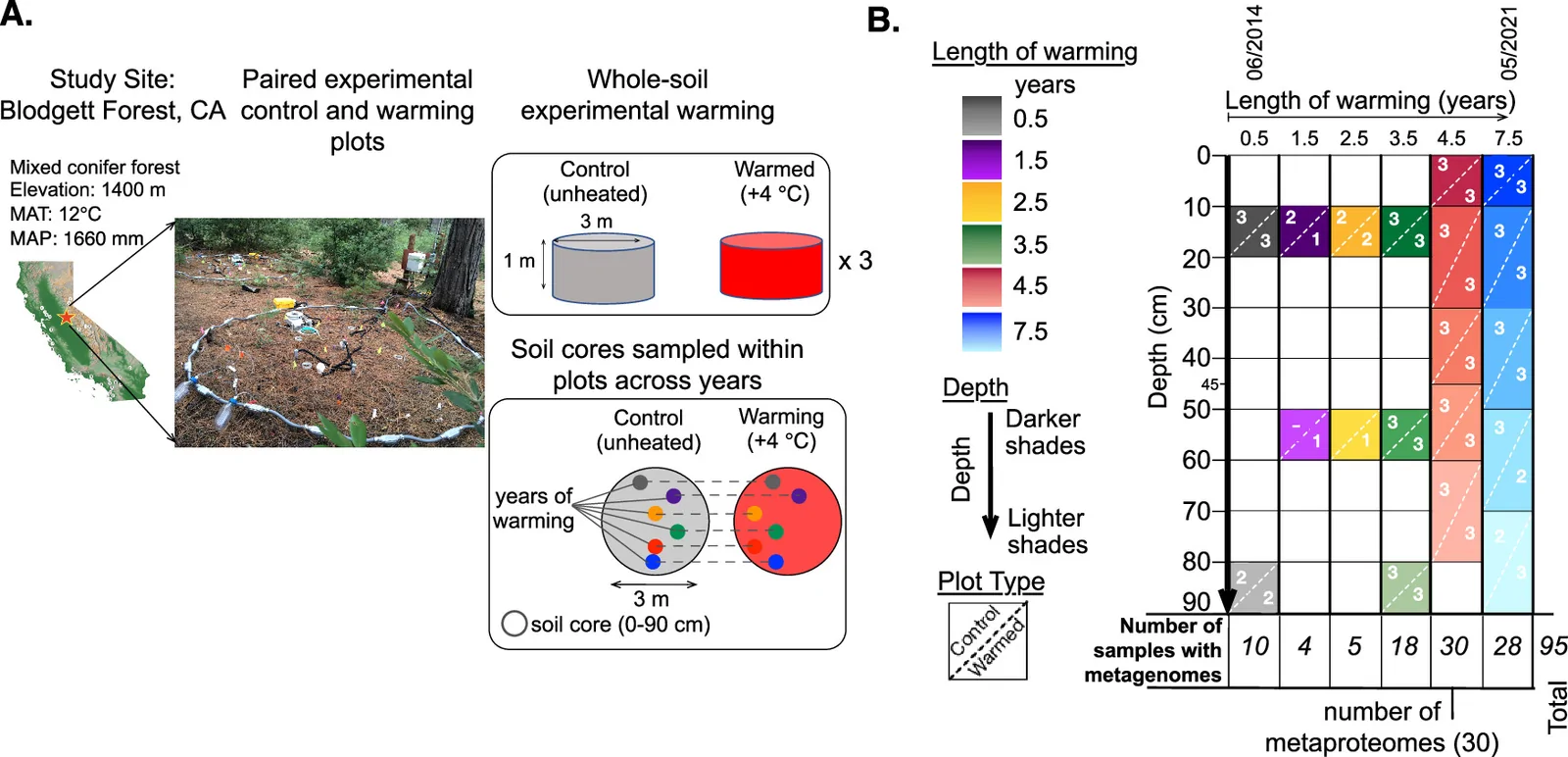
Experimental design and comprehensive proteogenomic sampling of the whole soil profile. We sampled the entire 0–90 cm soil profile from paired control and +4 °C warmed plots at Blodgett Forest over 7.5 years. Deep sequencing and co-assembly produced >2300 dereplicated MAGs, including many from uncultured phyla, creating an unprecedented genomic resource for deep soils. **A** Soil samples were collected from a whole-soil long-term warming experiment at the UC Blodgett Forest Research Station (Sierra Nevada, CA). The experiment involves three replicated paired control and warming plots (next to each other as shown in the picture), where the warming plots have been continuously +4 °C above ambient temperature from the surface down to 1 m. deep. The warming treatment at the site is still ongoing. Distinct soil cores from each plot were collected within each plot within a period of 7.5 years after the warming treatment had been started (refer to Supplementary Data 1 for sample-level details). **B** Schematic view of the analyzed data types for each of the depth intervals within a core across different years, which correspond to the length of warming. The colored boxes denote metagenome samples, with the number of samples from control and warmed plots successfully yielding nucleic acids. Each sampling year is denoted with a distinct color palette, with darker to lighter shades denoting the depth profile from most surface to the deepest. Metaproteome samples are from 2018, 4.5 years of warming, and from the same soil samples from that year. The total metagenome samples are reported (Supplementary Data 1), with additional information on the sample-level sequencing effort in Supplementary Data 2.

We collected soil samples from 0 to 90 cm depth in three paired control and warming plots at Blodgett Forest, CA, where the entire soil profile has been continuously warmed by +4 °C since 2014 (Fig. 1A). Over 7.5 years, 115 soil samples were collected after 0.5, 1.5, 2.5, 3.5, 4.5, and 7.5 years of warming. Ninety-five samples yielded high-quality metagenomes (Supplementary Data 1 and Fig. 1B), producing 3.61 Tb of raw data assembled into 56.9 Gb of contigs (≥1 kb; Supplementary Data 2 and 4, Supplementary Fig. 1). Deep sequencing and co-assembly approaches substantially expanded genome recovery, especially from later years with greater sequencing depth.

From these assemblies, we reconstructed 4888 metagenome-assembled genomes (MAGs) representing 2321 unique genomes (99% average nucleotide identity (ANI)), of which 1414 met high- or medium-quality standards^29^ (Supplementary Data 8). More than half (738) of these genomes were >70% complete with <10% contamination. These MAGs span 48 classes across 23 phyla, including many poorly sampled lineages (e.g., Gemmatimonadota, Dormibacteraceae, Binatia, Eremiobacterota, and DPANN archaea^30^). The abundance of closely related species varied markedly, suggesting responses are driven by environmental factors rather than phylogenetic constraints (Supplementary Fig. 2). This genome set greatly expands current references for studying microbial traits and carbon cycling in deep soils.

### Microbial communities are stratified by depth, with smaller and transient responses to warming

**Fig. 2:**
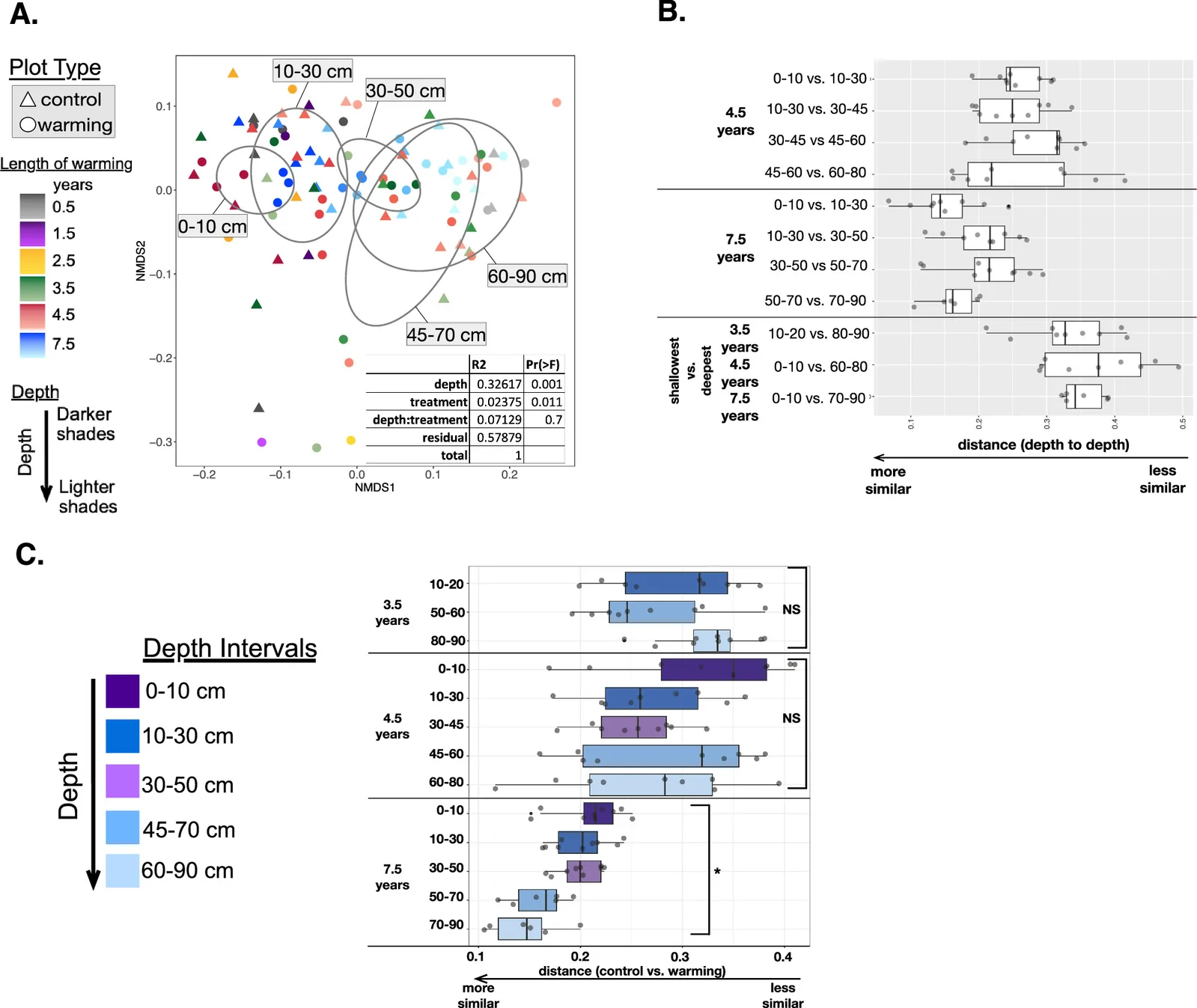
Dominant role of depth in shaping microbial community composition, with smaller, transient warming effects. Community analysis (rpsC census) showed depth as the dominant structuring force, explaining ~33% of variance compared to 2.3% for warming. The strongest turnover occurred around 30–50 cm, while warming effects were smaller, transient, and not conserved across taxa. **A** NMDS ordination of microbial communities (*n* = 95) from control and warming plots over 7.5 years, based on UniFrac distances of 3533 species groups (SGs) detected from all samples. The inset table shows variance partitioning by depth and warming. **B** and **C** show the microbial community distance comparisons stratified by combinations of factors for relevant pairs of communities (each point is for a distance between two communities). **B** Community shifts across consecutive (top two panels) and shallowest/deepest depths (bottom panel). Warming treatment length (4.5 and 7.5 years) microbial community distances between consecutive depths (top two panels) and between the most surface (shallowest) and deepest depths (3.5, 4.5, and 7.5 years) (bottom panel). **C** Community shifts with warming. Left panel: depth- and year-stratified microbial community distances between control and warming treatment. Color scheme refers to unified depth intervals as specified in the legend.

To characterize whole-community structure, we used a ribosomal protein (rpsC) census across assemblies, yielding 12,068 single-copy genes clustered into 3533 species groups (SGs) (Supplementary Data 5–7). Community ordinations (non-metric multidimensional scaling (NMDS) with weighted UniFrac distances) revealed significant effects of both depth and warming, but depth explained far more variance (33%) than warming (2.3%) (Fig. 2A and Supplementary Data 10). Community shifts in the control plots were sharpest between 30 and 50 cm (Fig. 2B), and communities converged between surface and deep soils over time (Fig. 2B bottom-most panel). Warming effects were weaker and declined with experiment duration: community divergence between control and heated plots decreased between 3.5 and 7.5 years (Fig. 2C). MAG-level responses to warming were more idiosyncratic than across depth, as few MAGs consistently responded across depths or years (Supplementary Figs. 3 and 4). Collectively, depth is the primary axis of community stratification, while warming exerts smaller effects diminishing over time.

### Extensive genomic potential for cycling of low-molecular-weight (LMW) and C1 compounds

Trait profiling of 2321 MAGs revealed broad capacity for low-molecular-weight (LMW) and C1 compound metabolism in addition to complex carbohydrate degradation (Supplementary Data 13). Methanol dehydrogenases were present in 620 genomes (27%) across 13 phyla, including Dormibacteria, Nitrospirota, Planctomycetota, Desulfobacterota_B, and Methylomirabilota, contrasting with Mediterranean grassland soils where representatives of these phyla with this capacity were absent^31^. Formaldehyde dehydrogenases (776 genomes) and formamidases (585 genomes) were common, linking methanol and methylamine turnover to formate production. Ureases (975 genomes) further linked C1 metabolism with nitrogen cycling.

The potential for inorganic nitrogen cycling was diverse: denitrification genes were distributed across multiple phyla, with nitrite and nitrous oxide reduction being particularly common. Nitrifiers (ammonia‑oxidizing archaea/bacteria (AOA/AOB), nitrite oxidizers) and dissimilatory nitrate reduction to ammonium (DNRA)-associated fermenters were also recovered, including from primer-recalcitrant lineages. Together, these genomes point to an overlooked capacity for coupled C1 and inorganic N turnover in deep forest soils.

### Depth selects for distinct resource acquisition and metabolic traits

Community-aggregated trait profiles (based on genomes and their abundances) revealed consistent depth associations across years (summarized in Table 1, Supplementary Fig. 4, and Supplementary Data 15). Near-surface communities were enriched in genes involved in methanol, formaldehyde, and formate oxidation, fermentation producing short-chain fatty acids, as well as cellular transport of oligosaccharides and amino sugars, inorganic nitrogenous compounds, amino acids, and organophosphorus compounds. In contrast, deep soils were enriched in genes involved in CO oxidation, fermentation producing alcohols, nitrogen fixation, and uptake of sugar alcohols. There was evidence for enrichment of H metabolism both in surface and deep soils, although the phylogenetic origin and diversity of NiFe-type hydrogenases differed.

**Table 1:** Depth-stratified genomic potential for microbial traits

A. Resource acquisition traits						
	Substrate assimilation		Substrate uptake		Substrate degradation	
Surface-enriched	• C1: formaldehyde oxidation (direct, glutathione, mycothiol pathways), formate oxidation, H4F pathway, PHB cycle, methanol oxidation • N: ammonia assimilation to glutamine • P: phosphatase, phytase • S: assimilatory sulfate reduction		• Carbohydrates: tri-/tetra-saccharide transporters, amino sugar transporters, carbohydrate acid transporters • Tricarboxylate transporters • N: nitrate, nitrite, nitrogen, urea, amide transporters • Aromatic acid transporters • Organophosphorus compound transporters • Free amino acid transporters • S compound transporters		• Complex carbohydrates degradation: cellulose (GH1, GH3, GH5, GH6, GH9, GH12), xylan and heteroxylan (GH39, GH43, GH54, GH62, CE3, CE4, CE6), xyloglucan (GH97), chitin (GH19, GH20, GH23, CE4), starch (GH13), pectin (CE12, CE13) • Simple compound degradation: ED pathway, sugar acids, protein degradation • Fatty acid and lipid degradation • Urea degradation	
Depth-enriched	• C1: CO oxidation, CO_2_ fixation, formaldehyde oxidation (THMPT pathway) • N: nitrogen fixation		• Carbohydrates: sugar alcohol transporters		• Complex carbohydrates: cellulose (GH44), xylan and heteroxylan (GH2, CE1), xyloglucan (GH4, GH27, GH36), chitin (GH18), starch (GH57), heteromannan (GH26) • Simple compounds: fructose, galactose • Methylamine degradation • Nitrile degradation to amide, ammonia	

B. Resource use traits						
	Chemolithoautorotrophy		Chemolithoheterotrophy		Chemoorganoheterotrophy	
Surface-enriched	• Hydrogenotrophy: nife-group-1 • N: DNRA (nirB) • S: sulfide oxidation (fccA, fccB), thiosulfate oxidation				• Aerobic respiration: ETC complex I (nuoA), ETC complex II (sdhC, sdhD), ETC complex III (fbcH), ETC complex IV (caa3-type, bo type, bd type) • Fermentation: pyruvate to short-chain fatty acids	
Depth-enriched	• Hydrogenotrophy: nife-group-2ade, nife-group-3abd, nife-group-4a-g • N: nitrite oxidation, DNRA (nrfA) • S: sulfide oxidation (sqr)		• N: denitrification (narG, norC, nosZ)		• Aerobic respiration: ETC complex I (nuoB), ETC complex IV (cbb3-type)	

C. Stress tolerance traits						
	Reactive oxygen species (ROS) scavenging	Protection, repair, degradation of denatured/misfolded proteins		Desiccation stress	pH stress	Oxygen limitation
Surface-enriched	• ROS scavenging enzymes	• ATP-dependent proteases		• Synthesis of compatible solutes • Transport of compatible solutes • EPS biosynthesis/export • Osmotic sensors, salt/potassium sensors	• Urease system • Cyclopropane fatty acid (CFA) synthase • Redox-sensing two-component systems • ATP-dependent proteases	
Depth-enriched						Oxygen-sensing two-component systems

MAG-based trait profiling revealed clear depth-dependent specialization: surface soils enriched in methanol, formaldehyde, and formate metabolism and diverse substrate uptake; deep soils enriched in CO oxidation, nitrogen fixation, alcohol fermentation, and uptake of simple sugars and sugar alcohols. The figure shows community aggregated genome-detected microbial traits (weighted by the coverage of the underlying genomes) that are significantly enriched in the surface and deep soils. The traits are organized under 3 trait dimensions (A. Resource acquisition traits, B. Resource use traits, and C. Stress tolerance traits), where subcategories were summarized at a level to show pathway-level differences across depth (for instance, amino acid utilization pathway and formaldehyde oxidation pathways). The comparisons for surface vs. depth were for control plots from treatment years matching 3.5, 4.5, and 7.5 years of warming, with a complete set of data from matched plots available (*n* = 3; refer to Fig. 1B).

These depth-enriched traits possibly reflect environmental gradients: plant-derived methanol and pectin-derived compounds near the surface, and CO production^32–35^ and nutrient scarcity at depth^36^. Depth-dependent H_2_ concentrations might have also selected for specific H_2_-oxidizing bacteria, as local H_2_ concentration and kinetics for NiFe-hydrogenase activity drive the energy potential of the H_2_ oxidation reaction^37^, and it is suggested that hydrogenases span a continuum of affinities^38,39^. Frequently observed trait co-occurrence (e.g., C1 metabolism with nitrogen transformations) suggests metabolic linkages central to carbon and nitrogen cycling through the soil profile (Supplementary Data 13).

### Long-term warming modifies trait potential, particularly near the surface

**Fig. 3:**
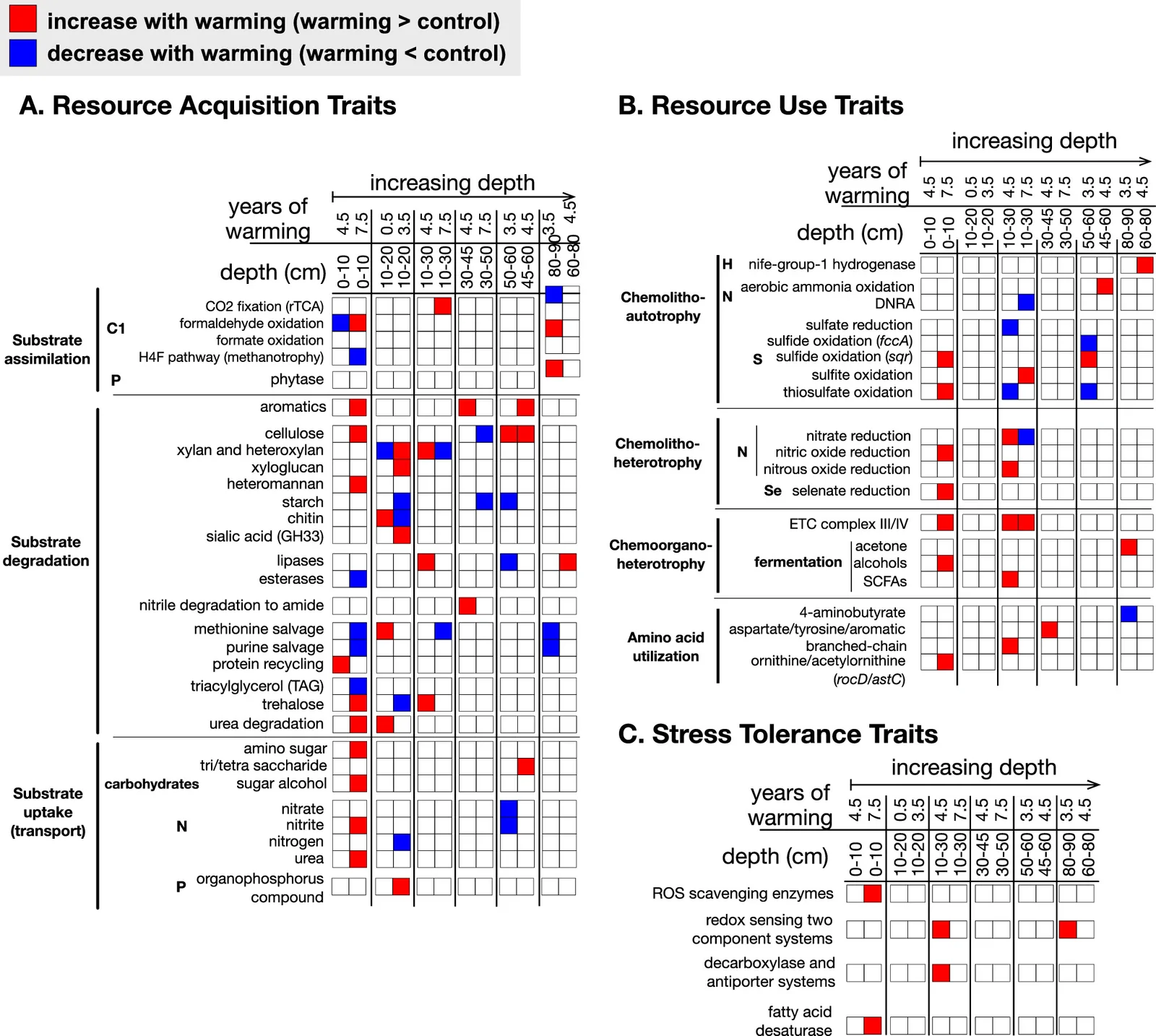
Depth- and time-specific impacts of long-term warming on microbial trait potential. Long-term warming altered a narrower but significant set of traits: **A** resource acquisition traits, **B** resource use traits, **C** stress tolerance traits. Surface soils showed enhanced uptake of specific carbohydrates classes (amino sugars and sugar alcohols), nitrogenous compounds, and expression of carbohydrate-active enzymes after 7.5 years. In contrast, responses in deeper soils were more variable. The figure summarizes community aggregated genome-detected microbial traits (weighted by the coverage of the underlying genomes) that are significantly different across control and warming plots (red/blue: increase/decrease with warming). Each comparison across control vs. warming plots was performed for the exactly matched depth intervals from years with a complete set of data from matched plots available (*n* = 3; refer to Fig. 1B), organized by increasing depth from left to right.

Compared with depth, warming altered a smaller subset of traits, and effects were often depth- and time-specific (Fig. 3 and Supplementary Data 15). After 7.5 years, surface soils showed increased potential for uptake of specific carbohydrate classes (amino sugars and sugar alcohols) and of organophosphorus and nitrogenous (nitrite and urea) compounds. Warming also increased the abundance of specific carbohydrate-active enzymes between 3.5 and 7.5 years of warming (e.g., those targeting cellulose, xylan and heteroxylan, heteromannan, xyloglucan, and sialic acids) in surface soils, while deeper soils showed more variable responses.

Warming affected traits involved in inorganic nitrogen turnover in complex ways: potential for nitric oxide reduction increased on the surface while nitrate and nitrous oxide reduction increased at mid-depth, albeit at different onsets with respect to warming length. Ammonia oxidation potential was enhanced deeper in the profile, whereas DNRA potential decreased. The potential for energy-generating processes, including aerobic respiration and fermentation, increased broadly, with depth-specific changes in fermentation end-products. Stress tolerance traits (for reactive oxygen species (ROS) detoxification, pH tolerance, and osmotic stress) were consistently enriched under warming, with depth-dependent patterns.

### Soil proteomics supports trait expression and reveals enzyme activities

**Fig. 4:**
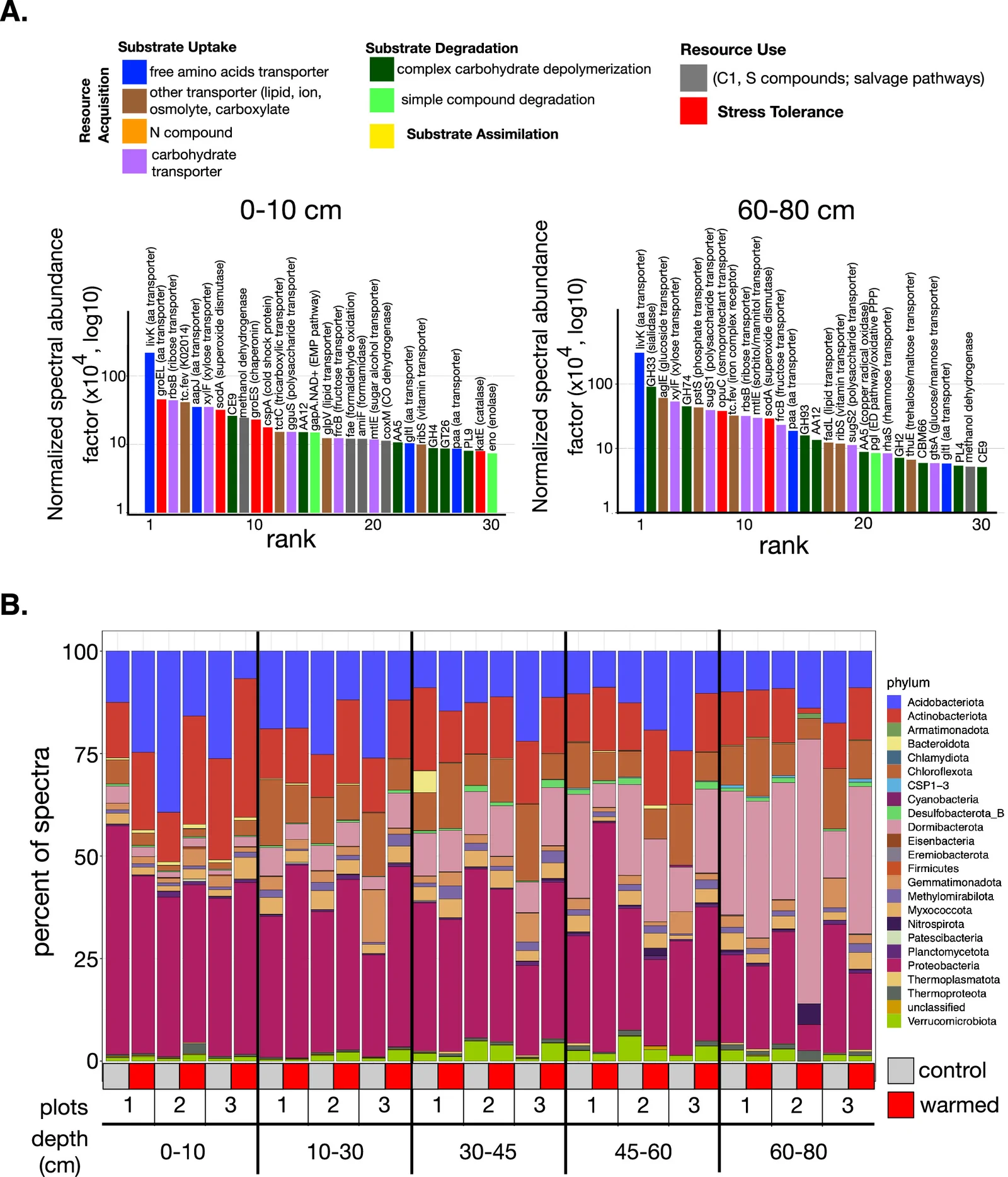
Proteomic validation of in situ microbial activity and discovery of novel glycan metabolism. Shotgun proteomics confirmed in situ activity of C1 cycling, nitrogen turnover, and stress responses. Depth-stratified protein expression mirrored genomic predictions: methanol oxidation was widespread, while formaldehyde detoxification, formate oxidation, CO oxidation, and ammonia oxidation showed depth- and warming-specific expression. Notably, sialidases and hemicellulose-active enzymes were abundant in deep soils, suggesting overlooked pathways in glycan metabolism. **A** Expressed protein families from all metagenome contigs ranked by their total normalized spectral abundances (log-scale) for surface and deepest soils for the top 30 families. For each protein family, the gene name and description are shown, with colors corresponding to the trait categories. **B** Relative expression of the reconstructed genomes as relativized total spectra assigned to each genome, summarized at phylum level, across all metaproteomics samples.

Proteomic analyses from 4.5 years of warming detected >25,000 proteins, including enzymes for C1 cycling, amino acid and sugar transport, and ROS defense (Supplementary Data 3 and 16–19). Expression profiles confirmed in situ activity of methanol/formaldehyde oxidation, formamidases, urea hydrolysis, and CO oxidation (Fig. 4A). Surprisingly, sialidases (GH33) and associated carbohydrate-binding modules (CBM40) were among the most abundant CAZymes in deep soils, suggesting unexplored roles for glycan metabolism.

Protein expression of C1 cycling enzymes decreased with depth, whereas carbohydrate-active enzymes, including ligninolytic and hemicellulolytic families, were more abundant in deep soils (Fig. 4A). Proteome depth profiles revealed stratified activity: Dormibacteria, Nitrospirota, and Verrucomicrobiota were more active at depth, whereas Proteobacteria and Acidobacteria dominated near-surface expression (Fig. 4B).

### Depth- and warming-specific expression of C1 metabolism and N cycling

**Fig. 5:**
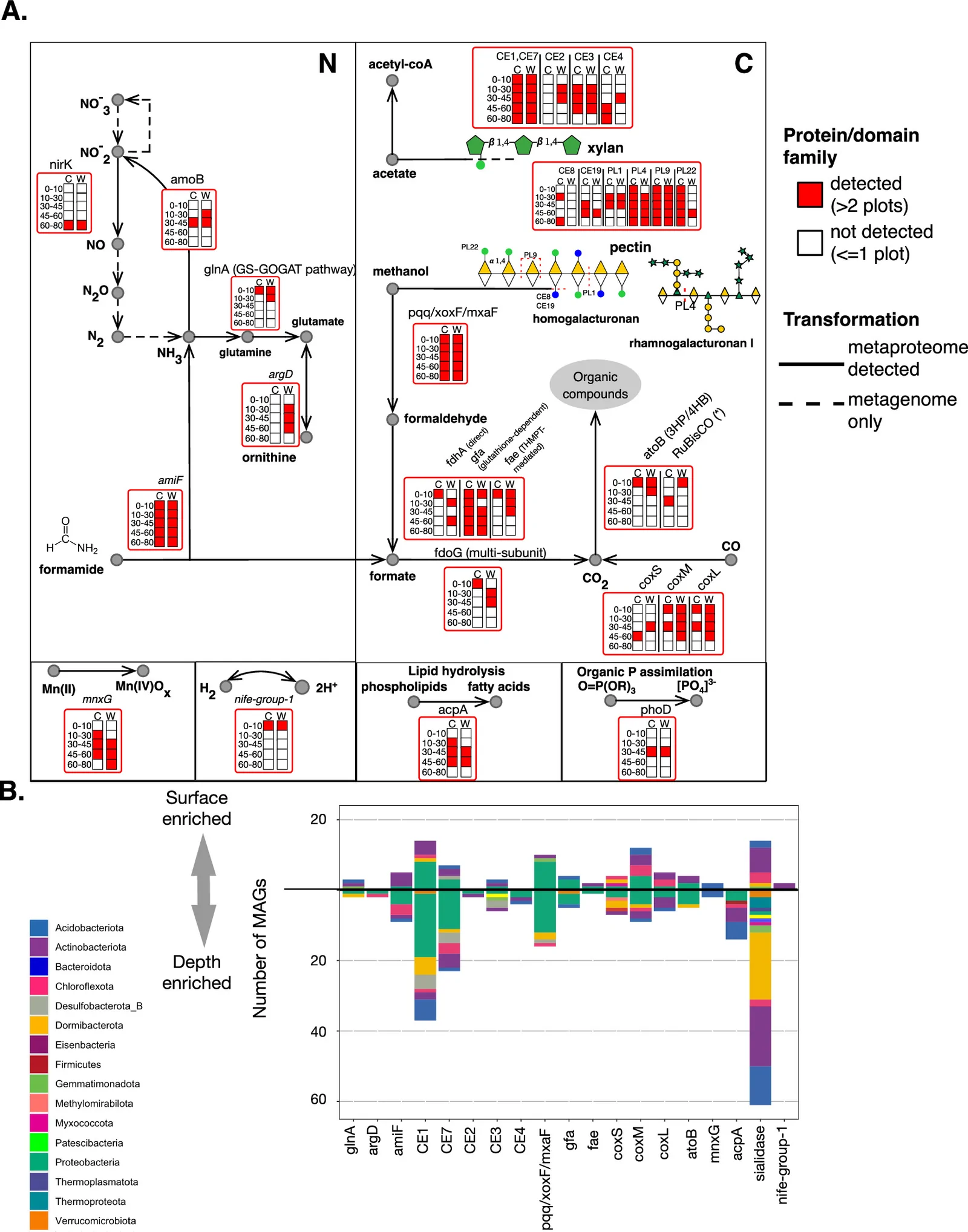
Mechanistic conceptual model of depth-dependent microbial trait regulation of carbon cycling under warming. Integrating genomic and proteomic data, we propose a model where depth-structured microbial traits, particularly C1 metabolism and nitrogen cycling, mediate soil carbon responses to long-term warming. Surface microbiomes respond rapidly via substrate uptake and stress tolerance, while deep-soil communities, enriched in novel lineages, show delayed but functionally distinct responses that modulate carbon–climate feedbacks. **A** Nutrient transformations with metaproteomic evidence across the depth profiles from control and warming plots. For each transformation and protein family, the red filled squares denote detection of at least two spectra at orf level and from at least two replicate plots. Top left and right panels show nitrogen (N) and carbon (C) transformations, respectively. Broken lines for inorganic N species depict metagenome-detected transformations with no detected proteins. RuBisCO-mediated C fixation (*) was detected in only one replicate plot. **B** Distribution of surface- and depth-enriched genomes and their lineage, with evidence of expression for the protein families catalyzing transformations in (**A**).

Targeted analysis of replicated plots highlighted warming-induced stimulation of formaldehyde detoxification and formate oxidation in mid-depth soils (10–60 cm), and increased CO oxidation down to 60 cm (Fig. 5A). Formamidase activity was detected across all depths, linking C1 and N cycles. Ammonia oxidation was expressed in both control and warmed plots at mid-depths but showed warming-specific enhancement near the surface.

Hydrogen metabolism was most active in surface soils, while manganese oxidation occurred below 10 cm and the shallowest depth in which it was found shifted deeper in heated plots. Many of these traits were encoded in rarely sampled deep-soil lineages (e.g., Dormibacteria, Gemmatimonadota, and Thermoplasmatota) (Fig. 5B), suggesting adaptation to depth-dependent resource gradients.

## Discussion

Soils represent the largest terrestrial carbon reservoir, yet the microbial processes governing their responses to climate warming remain poorly understood. By combining genome- and proteome-resolved analyses across a whole-soil warming experiment, we show that microbial traits are strongly stratified across depth and that their responses to 4 °C warming, though less prominent than the depth effect, are functionally consequential. More than 2300 dereplicated genomes, spanning 23 phyla and including many uncultured lineages, reveal a microbial trait landscape that differs sharply between surface and deep soils. Moreover, proteomic data confirm that many of these traits are expressed in situ. Together, this work demonstrates that subsoils, long overlooked in field studies and biogeochemical models, harbor metabolically distinct and functionally active microbial communities that potentially mediate how carbon is stabilized or lost under long-term warming.

Soil depth was the primary axis of microbial trait selection and activity. Depth explained ~14-fold more variance in community structure than did 4 °C of warming (33% vs. 2.3%), underscoring its role as the dominant ecological filter. The sharpest shift associated with depth-only occurred between 30 and 50 cm, aligning with steep physicochemical gradients in SOM chemistry, root inputs, and redox conditions^8,13,40^. This stratification was mirrored in traits, with surface microbiomes specialized in methanol, formaldehyde, and formate metabolism (encoded in over 40% of MAGs), consistent with high near-plant methanol fluxes and widespread soil/phyllosphere methylotrophy^41–44^. In contrast, deep microbiomes had higher CO oxidation, nitrogen fixation, and alcohol fermentation capabilities, aligning with evidence that subsurface microbes can persist via atmospheric CO oxidation and that oxygen and other more favorable substrates become limiting with increasing depth^45–49^. Proteomic depth profiles generally confirmed these patterns, with CO oxidation and nitrogen fixation peaking below 50 cm. However, methanol oxidation was consistently active across depths, consistent with our observations of high micromolar concentrations of methanol in soil water extracts across depths (Alves et al., unpublished).

The enrichment of GH33 sialidases and CBM40 modules in deep soil microbiomes and evidence for their protein expression suggests that these enzymes may facilitate the breakdown of glycoproteins or glycolipids, including those associated with microbial necromass. By cleaving terminal sialic acids, known to protect glycoproteins from proteolytic degradation and thermal denaturation^50^, microbial sialidases may destabilize these glycoconjugates and unlock previously inaccessible carbon and nitrogen reservoirs in subsoil environments. Although common in host-associated microorganisms owing to the secretion of host mucin-associated glycoproteins^51^, we believe this is the first evidence of their expression in subsoils, challenging the prevailing view that glycoprotein-rich necromass may constitute a largely stable soil carbon pool^52^. The metabolism of the analyzed soil microbes is tightly coupled to both plants and soil fungi. In surface soils, microbial communities were enriched in traits for metabolizing direct plant inputs, such as plant-derived methanol and pectin from root exudates and litter. However, warming-induced declines in fine root mass^40^ likely exacerbate carbon limitation in the subsoil, restricting plant-derived substrate availability and driving deep-soil microbes toward alternative metabolisms like C1 and CO oxidation. The highly expressed sialidases in deep soils indicate that these bacteria actively scavenge glycoprotein-rich microbial and fungal necromass, unlocking carbon and nitrogen reservoirs possibly generated by fungal turnover. Collectively, these results highlight that deep-soil microbiomes are not substrate-limited, but rather harbor distinct metabolic strategies, expanding the known scope of microbial contributions to SOM turnover.

Although warming explained relatively little variance in overall microbiome composition, it altered trait distributions in ways that were both depth- and time-specific. At the surface, long-term warming enhanced uptake of amino sugars, sugar alcohols, and nitrogenous compounds, expanded carbohydrate-active enzyme repertoires (e.g., cellulases, sialidases), and enriched stress tolerance traits such as ROS detoxification. These patterns are consistent with rapid microbial adjustments to faster substrate turnover and environmental fluctuations^14,27,53^. In contrast, deeper soil microbiomes showed more variable, but functionally critical responses: stimulation of formaldehyde detoxification, formate oxidation, and CO oxidation was observed in mid-depth soils (10–60 cm), whereas inorganic nitrogen transformations showed bidirectional shifts, namely a decline in DNRA potential and higher ammonia and nitrous oxide turnover increased at depth. Importantly, warming effects on community composition weakened over time but increased on trait expression, suggesting that functional plasticity plays a greater part in the microbial functional response to warming than taxonomic turnover.

Strikingly, uncultured lineages were prominent in encoding and expressing key functions in these soils. Dormibacteria, Gemmatimonadota, and Thermoplasmatota collectively contributed hundreds of MAGs, many near-complete, and were disproportionately associated with C1 metabolism and nitrogen cycling. Proteomic data confirmed their in situ activity, particularly in deep soils where Dormibacteria and Nitrospirota expression increased while Proteobacterial and Acidobacterial activity declined. These lineages not only expand the phylogenetic breadth of soil genomes but also highlight that “hidden” taxa may play outsized roles in regulating soil–climate feedbacks. Their functional contributions, largely invisible to amplicon-based primer-dependent surveys, underscore the value of proteogenomic approaches for capturing the true scope of soil functional potential^54–56^.

Warming generally increases the energetic costs to maintain cellular viability, diverting resource allocation away from growth toward survival^57,58^. In this field warming experiment, we previously measured the response of microbial biomass to warming and have previously shown that the response varies between the topsoil and subsoil, with some dependency on the measurement method used^59^. After 4.5-year of whole-soil warming, bulk measurements of microbial biomass carbon showed no significant change with warming at any soil depth^59^. However, targeted molecular analyses proved more sensitive at capturing the treatment effect^59^. Using phospholipid fatty acid (PLFA) concentrations, a more precise proxy for living microbial biomass, we found that microbial biomass decreased by 28% in the subsoil (below 30 cm) but remained unaffected in the topsoil^59^. Similarly, concentrations of branched glycerol dialkyl glycerol tetraethers (brGDGTs), which could be considered indicators of microbial necromass, were also lower in warmed subsoils between 50 and 90 cm^59^. This pronounced decrease in subsoil microbial biomass is likely driven by carbon limitation; the warming treatment reduced fine root mass^40^ and depleted unprotected soil organic carbon^60^, depriving subsoil microorganisms of easily accessible organic matter^59^. Our genome-resolved data provide opportunities for characterization of biomass changes of specific microbial strains or functional groups we report here through qPCR or ddPCR assays by allowing precise, custom primer design targeting uncultured microorganisms. Such measurements would strengthen the interpretation of CO₂ flux patterns and carbon use efficiency, a crucial factor controlling soil carbon retention under warming conditions^61–63^.

Our results demonstrate that warming affected inorganic nitrogen dynamics traits. These patterns may suggest a transition toward N-removal pathways that usually occur under low soil C:N conditions and when carbon is relatively limiting. Previously, we showed that while SOC and total nitrogen concentrations were significantly lower in the warmed subsoils (below 20 cm), the soil C:N ratios did not significantly differ between the control and warmed plots at any soil depth^40,60^. Therefore, the observed functional shifts in nitrogen cycling do not appear to be driven by a warming-induced shift toward lower bulk soil C:N ratios. On the other hand, our previous data well support relative carbon limitation as a driver of increased microbial N-removal processes under warming. Mediterranean forest soils are generally considered carbon limited^28^, and this limitation increases with depth^64^. During our experiment, sustained warming likely exacerbated this carbon limitation over time, as evidenced by a pronounced depletion of easily accessible, unprotected free particulate organic matter in the warmed subsoils^28,60^.

Our findings provide a mechanistic framework for understanding depth-dependent carbon cycling under warming. Surface soils respond rapidly through shifts in community composition and substrate uptake capabilities, while deep soils respond more slowly but with functional traits related to alternative metabolisms, namely CO oxidation, C1 transformation, and nitrogen fixation, which may sustain respiration and greenhouse gas fluxes over longer timescales. Beyond these conifer forest soils, we note that our proteogenomic results align with broad evidence that subsurface microbes persist via atmospheric CO oxidation, alcohol fermentation, and other alternative metabolisms as more favorable substrates and oxygen become limiting with depth^45,46,49^. This framework could help explain the sustained ~30–37% increase in soil respiration observed at Blodgett Forest more than 7 years of warming^27^. From a modeling perspective, even though the importance of microorganisms for global biogeochemical cycles is well recognized, efforts to explicitly incorporate them into Earth System Models have only begun in recent years^65–69^. The next generation of ecosystem models is poised to more heavily incorporate trait-based approaches to explicitly model microbial control of organic matter decomposition^65–67,70^, which will necessitate incorporation of microbial traits that are demonstrated to enhance fitness in deeper soils, as well as accounting for their substrates and metabolic/biogeochemical pathways.

Our study stresses that soil responses to environmental change cannot be fully understood without accounting for the depth-stratified microbial traits that govern carbon transformations. For the case of soil warming, surface microbiomes respond rapidly through enhanced substrate uptake and stress tolerance, while deep-soil communities, structured by strong physicochemical filters and dominated by uncultured lineages, contribute slower but functionally distinct responses centered on C1 metabolism and nitrogen turnover. Together, these depth-dependent processes provide a mechanistic basis for sustained soil respiration under long-term warming and emphasize the urgent need for models that move beyond topsoil-centric perspectives and incorporate microbial trait variation across the whole soil profile.

## Methods

### Study site and field soil warming experiment

The study site is located in the University of California Blodgett Experimental Forest in the Sierra Nevada foothills of California at 1370 m above sea level (120°39′40′′W; 38°54′43′′N)^27^. The average yearly precipitation at the site is 1660 mm, with most falling as snow between November and April. The mean annual air temperature is approximately 12.5 °C. The site is in a thinned mixed conifer forest with dominant overstory species of ponderosa pine (Pinus ponderosa), sugar pine (Pinus lambertiana), incense cedar (Calocedrus decurrens), white fir (Abies concolor), and Douglas fir (Pseudotsuga menziesii). The soil order at the site is classified as Alfisol of granitic origin with a developed O horizon as part of the Holland-Bighill complex.

The field warming experiment, described in detail in Hicks Pries et al. and in its Supplementary Methods^27^, started in October 2013 when the heaters were turned on. Shortly, the experimental design includes three pairs of heated plots and unheated control plots, each circular with a 3 m diameter, and each pair of heated and control plots situated next to each other. The coordinates corresponding to each of the three paired plots are given in Supplementary Data 1. Plots within a pair are adjacent and located in the same stand conditions to minimize differences in overstory, slope, and soils, with the control plot providing the local reference for the heated one. The distances between the 3 paired experimental plots are 21, 25, and 34 m. Each plot is circular, 3 m in diameter. To minimize the effect of disturbance across different years, soil cores were taken between heating rods and away from temperature probes, maintaining the integrity of the heating system and the monitoring instrumentation, and avoiding repeated sampling in the same plot regions. Heating treatment followed the design of Hanson et al.^71^ with resistance heating cables (BriskHeat, Ohio, USA) that warm soil by 4 °C above ambient temperature down to 1 m depth, while preserving the natural temperature gradient through the soil profile. Unheated cables were placed in the paired control plots using the same design. Soil temperature and soil volumetric water content were monitored continuously across the depth profile using temperature sensors (Omega 44005) and enviroSCAN (Sentek, Australia) probes. These measurements were used as input to a system that drives the power to the heaters in order to maintain the targeted 4 °C warming in the paired heated plots (see Hicks Pries et al.^27^ and Soong et al.^60^ for details).

### Soil sampling, DNA extraction, and metagenome sequencing

We collected soil cores from each plot using a 5-cm diameter AMS corer (AMS Inc., Hayward Falls, ID, USA) at six distinct time points: 0.5, 1.5, 2.5, 3.5, 4.5, and 7.5 years after the warming treatment began. The soil depth intervals spanned from the surface to 90 cm deep. The exact intervals changed across sampling years due to field practicalities. Within each field season, the same depth intervals were used for all plots. Supplementary Data 1 lists the depth intervals for all the samples. Single-use sterilized polycarbonate core sleeves were used to protect the soil cores. Soil samples were homogenized in sterile bags and were immediately placed on dry ice and stored at −80 °C DNA extraction. For each sample, we extracted DNA from 0.25 to 0.5 g of field-moist soil using the DNeasy PowerSoil Kit (Qiagen, Germantown, MD, USA). For 2014, 2015, and 2021 samples, metagenomic library preparation and DNA sequencing were performed at the Joint Genome Institute. Samples from 2016 and 2018 were sequenced at the UCSF Center for Advanced Technology as described by Dove et al.^28^. A total of 101 metagenomes were sequenced from 95 samples. Six samples were sequenced twice using JGI’s regular DNA with 270 bp fragment and low input DNA with 400 bp fragment protocols. Refer to Supplementary Data 2 for metagenome metadata and statistics.

### Metagenome assembly

Metagenomic reads from each sample were assembled following the JGI metagenome assembly pipeline^72^. In addition to single-sample assemblies, samples from 2016 and 2018, which had shallower sequencing depth, we also performed co-assemblies by pooling samples from the replicate plots by treatment and depth interval (refer to Supplementary Data 4 for all performed assemblies). Briefly, raw reads from each assembly were subjected to quality trimming, artifact removal, linker and adapter trimming, and spike-in removal using BBTools RQCFilter (parameters: mlf=0.333 maxns=3 removemicrobes=t minlength=51 rna=f removecat=t removehuman=t clumpify=t minavgquality=3 trimq=0 removemouse=t phix=t qtrim=r trimfragadapter=t khist=t removedog=t barcodefilter=f sketch=f mapk=13). The resulting reads were corrected for sequencing errors using BFC (Bloom filter correction with parameters: −1 -k 21 -t 10). Orphan single-end reads were removed from the paired-end library using the Seqtk dropse subcommand (https://github.com/lh3/seqtk). The final reads were assembled into scaffolds using metaSPAdes with parameters: --only-assembler -k 33,55,77,99,127 --meta -t 32 -m 2000. For each assembly, scaffolds shorter than 1 kbp were removed using SeqKit^73^ (seqkit seq --min-len 1000).

### Metagenome open reading frame prediction

Open reading frames (ORFs) were predicted in all scaffolds using Prodigal (v2.6.3) in metagenome mode with default translation table (-g 11) and options -m -p meta. The outputs for the nucleotide and protein translations were saved with options -d and -a, respectively.

### rpsC marker gene identification and clustering to define species groups and abundance quantification of species groups

Species groups (SGs) were defined based on the single-copy phylogenetic marker gene for 30S ribosomal protein S3^31^. rpsC (rpS3) marker protein sequences were identified from all translated open reading frame sequences using the custom HMM (hidden Markov model) from Diamond et al.^31^. (https://raw.githubusercontent.com/SDmetagenomics/Diamond_NatMicro_2019/refs/heads/master/HMMs/rpS3/rpS3_Diamond2019.hmm) with the built-in specified trusted cutoff (TC = 40). From all assemblies, we detected 12068 rpsC sequences (Supplementary Data 5). The detected sequences were clustered at 99% sequence identity (-cluster_fast -sort length -id 0.99 -maxrejects 0 -maxaccepts 0) with USEARCH (v8.1.1861_i86linux64)^74^ to generate 3536 clusters. Three non-prokaryotic clusters were removed (rpsC_lf.cluster3429, rpsC_lf.cluster1061, rpsC_lf.cluster1736), leaving 3533 clusters, which we used to represent species groups. For each rpsC protein cluster, the longest scaffold was identified and used for read mapping to quantify abundance for the cluster across all samples (Supplementary Data 5). The abundance of rpsC clusters were profiled across samples by mapping the reads from each metagenome to the set of longest scaffolds associated with the species groups using Bowtie2^75^ with the sensitive preset option (--sensitive). Coverage statistics were calculated per scaffold using CoverM^76^ contig command with the following options: “proper-pairs-only,” “min-read-percent-identity = 0.99,” “min-read-aligned-percent = 0.75.” Mean coverage matrix (scaffolds × metagenomes fastqs) from CoverM output (-m mean) was normalized for sequencing depth for metagenome as follows: mean_norm_coverage = mean_coverage/total sequenced bp from sample*10E8 (Supplementary Data 7). For six samples with two metagenomes each, the mean normalized coverage from two metagenomes was assigned to the sample.

### Phylogenetic inference and taxonomic assignments for species groups

3533 translated rpsC proteins representing the species groups identified were concatenated with taxonomy labeled reference rpsC sequences from Hug et al.^77^ in a single fasta file. The sequences were aligned with MAFFT (v7.427)^78^ with default options (second progressive alignment method FFT-NS-2), and the resulting multiple sequence alignment (MSA) was trimmed with trimAl^79^ with option (-gapthreshold 0.05). A phylogenetic tree was inferred from the trimmed MSA with FastTree with default options (JTT+CAT model)^80^. For each species group, phylum, and class level, lineages were assigned based on their relative position with respect to the reference sequences in the phylogenetic tree (Supplementary Data 6).

### Microbial community structure analysis, variance partitioning

All analyses of microbial communities were based on the species groups (SGs) defined with the rpsC marker gene from metagenome assemblies, and performed with the R packages vegan^81^, phyloseq^82^, dplyr^83^, tidyr^84^, tibble^85^, ggplot2^86^. SG mean sequencing depth normalized coverage were Hellinger standardized to relativize species abundances (vegan::decostand, method = “hellinger”). SGs with a coefficient of variation >3 or with <5 samples with raw coverage >= 0.5 were removed, yielding 2016 SGs. A phyloseq object was built from the corresponding Hellinger standardized coverage table and the phylogenetic tree. Weighted UniFrac distance between microbial communities was calculated (phyloseq::distance; method = “unifrac,” weighted = TRUE). The communities were NMDS (non-metric multidimensional scaling) ordinated using vegan::metaMDS function (*k* = 3, try = 500, trymax = 500; NMDS stress value = 0.095). Estimates of the weighted covariance matrix and the mean of the data were calculated with cov.wt base function in R. The corresponding covariance ellipses for each consolidated depth interval (Supplementary Data 1) were calculated with the vegan::veganCovEllipse function. Ordinations and covariance ellipses around the consolidated depth intervals were visualized using the first two NMDS dimensions (Fig. 2A). Variance partitioning of the distance matrix across depth and warming treatment was performed with a permutational multivariate variance partitioning procedure, as implemented in vegan::adonis2 function (model: weighted_unifrac_distances ~ depth ×  treatment, by = “terms”) (Supplementary Data 10). The distance matrix was transformed into long format (tidyr::pivot_longer), the metadata for the matched samples were appended, and the distances for pairs of communities with the described constraints specified in Fig. 2B, C legend were selected for plotting distributions.

### Genome binning, aggregation and dereplication

Scaffolds longer than 1 kbp from metagenomes assemblies were binned into draft genomes using three metagenomic binning programs and aggregated into consensus bins. Coverage profiles for each sample were measured across samples grouped into binning groups, as specified in Supplementary Data 2. Given that the depth variation was the largest among variables, the binning groups were chosen to maximize expected variance. For each metagenome assembly, reads from samples in the binning group were mapped to the scaffolds longer than 1 kbp using Bowtie2^75^ with the sensitive preset option (--sensitive). The differential coverage profile for each scaffold across the respective samples was used as input to (1) COCACOLA^87^, (2) MaxBin2.0^88^, (3) MetaBAT2^89^. The highest quality bin from each assembly was chosen via aggregation with DasTool^90^. The resulting 4888 bins from all assemblies were pooled together. Bin completeness and contamination were computed using CheckM^91^, and bins passing completeness >50%, contamination <25% were dereplicated using dRep^92^ (2321 bins, Supplementary Data 8). Bin representativeness of the community was assessed by associating rpsC clusters with genome bins (Supplementary Data 9, Supplementary Figs. 6 and 7).

### Genome coverage and relative abundance calculation across samples

To calculate sample-specific MAG coverage and relative abundances, MAGs were profiled across samples with the CoverM^76^ genome command with the following options: “--mapper minimap2-sr” (default mapper), “--discard-unmapped,” “--min-read-aligned-percent 0.75,” “--min-read-percent-identity 0.95,” “--min-covered-fraction 0,” “--discard-unmapped,” “--methods relative_abundance trimmed_mean.” The relative abundance matrix from CoverM output (relative_abundance) was normalized to the total percent mapped reads from each respective sample to create a normalized relative abundance matrix. Mean coverage matrix from CoverM output (trimmed_mean) was normalized for sequencing depth as follows: mean_norm_coverage = trimmed_mean/total sequenced bp from sample*10E8. For six samples with two metagenomes each, the mean normalized relative abundance or coverage from the two metagenomes was assigned to the sample. The resulting two data products, normalized relative abundance matrix and normalized mean coverage matrix, each of dimensionality “number of MAGs × number of samples,” were used in downstream differential abundance analysis.

### Microbial genome trait annotation

For the 2321 genome bins passing completeness and contamination thresholds, gene loci were called using Prodigal (v2.6.3)^93^. Genomes were mapped to the trait hierarchy from microTrait^94^ with an HMM-based workflow. For each genome, the gene content was profiled based on searches of the genes against HMMs (Hidden Markov Models) selected from primarily from microTrait^94^ but also from KOfam^95^ (https://www.genome.jp/ftp/db/kofam/), METABOLIC^96^, Diamond et al.^31^, and dbCAN (v6)^97^. For detailed information on HMM information, the associated protein families, and the trait assignment based on HMM profiles, refer to Supplementary Data 12. The HMM searches were performed using HMMER3^98^ hmmsearch (v3.2) command. For full-length gene models, the trusted cutoffs built into the models were used to filter the hits (hmmsearch --domtblout --cut_tc) by score. The resulting hits from domtblout files were further filtered to remove hits that didn’t cover at least 60% of the HMM length. For CAZy domain hits from dbCAN, the filtering was performed with hmmsearch-parser.sh from dbCAN. This filtering corresponds to picking the domain with the lower *E*-value if multiple domains overlapped the same region of sequence, and using an *E* < 10E-5 if the alignment >80 aa; otherwise *E* < 10E-3 and HMM coverage >30%.

### Calculation of community aggregated traits

For each sample, community aggregated traits were calculated using MAGs based on their trait profiles and their normalized relative abundances in the respective sample (Supplementary Data 14). For each trait, the total community aggregated value for a sample was calculated by summing the relative abundances of the MAGs in that sample carrying the trait. For count traits (i.e., when the copy number was taken into account), the relative abundances were weighted by the trait count per genome.

### Differential abundance analysis for genomes and community aggregated traits

Differential abundance analysis of MAGs and community-aggregated traits between control and warming plots (treatment effect) and sampling depth (depth effect) was determined using normalized mean coverage matrix (for MAGs) and community-aggregated trait values (for traits) as the input for linear mixed-effects models, as implemented in the nlme R package^99^ (Supplementary Data 11 and 15). To test warming treatment effect, a separate model was built for date and depth matched samples from control and warming plots for which data from all three replicates was available (refer to Fig. 1B; 12 models for 10–20 cm from 06-2014, 10 to 20 cm from 05-2016, 50 to 60 cm from 05-2016, 80 to 90 cm from 05-2016, 0 to 10 cm from 06-2018, 10 to 30 cm from 06-2018, 30 to 45 cm from 06-2018, 45 to 60 cm from 06-2018, 60 to 80 cm from 06-2018, 0 to 10 cm from 05-2021, 10 to 30 cm from 05-2021, 30 to 50 cm from 05-2021). Depth effect was tested using samples from control plots and for years for which data from all three replicates were available across the depth profile (refer to Fig. 1B; 2016, 2018, and 2021). Each model was built using restricted maximum likelihood with replicate plot (*n* = 3) as a random effect to test the effect of warming treatment on the normalized genome coverage or community aggregated trait values. For warming treatment effect, models were fit with the following form: y  ~ treatment, random = ~1|replicate, method = “REML,” where y was either MAG coverage or trait value and treatment was a categorical variable with either control or heated. For depth effect, models were fit with the following form: y ~ depth, random = ~1|replicate, method = “REML,” where y was either MAG coverage or trait value and depth was a categorical variable with the depths from the respective year. Model slope *p-*values were extracted, and significance was tested at alpha = 0.05.

### Metaproteomics methods and peptide mapping

Samples from soil cores collected after 4.5 years of warming from control and warming plots were selected for metaproteomics analysis (30 samples = 3 replicates × 2 (control/warmed) × 5 depth intervals), performed at the Environmental Molecular Sciences Laboratory (EMSL) at Pacific Northwest National Laboratory (PNNL). For soil protein extraction, we employed an amino‑acid–blocking soil protein extraction protocol to minimize sorption losses to minerals. Soils were pre‑incubated with a mixture of free amino acids to saturate mineral adsorption sites before in situ detergent lysis and proteome recovery, as previously described in ref. ^100^. Following extraction, proteins in the soil extracts were digested, purified, and analyzed by liquid chromatography-tandem mass spectrometry (LC-MS/MS) as in ref. ^100^. Tandem mass spectrometry (MS/MS) spectra from all LC-MS/MS datasets were exported as ASCII text (.dta format) using MSConvert (http://proteowizard.sourceforge.net/tools/msconvert.html), and the resulting datasets were searched following a target-decoy approach^101^.

The following target-decoy search strategy was implemented for protein identification. An extensive protein sequence database capturing all the sequence diversity at the experimental site was constructed by pooling the translated predicted ORFs from all the assembled scaffolds. The final list was dereplicated at 100% amino acid identity. The final dereplicated database contained ~76 M proteins (~16,000 M amino acids) within ~27 M scaffolds (>1 kb) from 115 assemblies. The database was split into 3 manageable size databases (~25 M sequences). Each database was searched with MS-GF+ using PNNL’s DMS processing pipeline (https://github.com/PNNL-Comp-Mass-Spec/DMS-Analysis-Manager) with a lenient cutoff of 5% to extract a reduced set of proteins for a single database that was used for final matching of all samples together. Raw search results were filtered at 1% FDR at the peptide level, estimated by the target-decoy approach^101^. A parsimony approach was used to infer proteins from the peptides identified^102^, with one unique peptide required for a positive identification of a protein.

The filtered hits from MS2 spectra from individual samples were summarized as a spectral count matrix (orfs × samples). There were 122,374 orfs with at least one spectral count across 30 samples, so the dimension of the final spectral count matrix was 122,374 × 30.

### Limitations of proteogenomics approach

Interference from extracellular/relic DNA for metagenomics and extraction efficiency for metaproteomics are limitations of soil proteogenomics techniques. Our genome, rather than read-centric, analysis workflow remedies the effect of extracellular DNA by requiring the functional traits to be detected within genomes rather than within individual reads recruited to a database of sequences. While we have no direct evidence from our study, nor do we know of any direct empirical evidence, fragmented DNA has previously been linked to poor assembly outcome^103^. Our expectation is that relic DNA results in assembly errors (de Bruijn graph breakage) that reduce the recovery of quality MAGs. While extracellular DNA might have inflated apparent functional potential, extraction efficiency for proteomics limits detection sensitivity.

In soils, a substantial fraction of proteins becomes tightly associated with clay minerals, Fe/Al oxides, and mineral-associated organic matter, and extraction tests with radiolabeled proteins indicate that even strong extractants fail to recover the full mineral-bound pool^104,105^. This suggests that proteins from non-living cells (relic proteins) are less likely to contribute to our proteomic observations. Furthermore, during extraction we used an amino‑acid-blocking soil protein extraction protocol^100^, which reduces sorption of cellular proteins to minerals^106^. Therefore, our metaproteomic data should be interpreted as representing the cellular soluble and weakly sorbed subset of the soil proteome. With this in mind, it is theoretically possible that some of the observed protein expression differences could be attributed to variable protein sorption due to mineralogical differences through the soil profile.

### Reporting summary

Further information on research design is available in the Nature Portfolio Reporting Summary linked to this article.

## Supplementary information

Reporting Summary

Supplementary Information

Description of Additional Supplementary Files

Supplementary Data 1–19

Transparent Peer Review File

## Source data

Source Data

## Data Availability

Metagenomics sequencing data for each sample are available at the links provided for NCBI BioProject accession/NCBI Biosample accession IDs listed in Supplementary Data 2. Metagenome-assembled genomes and associated metadata are available on the Environmental System Science Data Infrastructure for a Virtual Ecosystem (ESS-DIVE) repository (Karaoz et al. https://doi.org/10.15485/2588706). Raw metaproteomic data acquisitions from Mass Spectrometer are available in EMSL Science Central under EMSL project ID 50964. The data products from MS-GF+ searches are deposited in ProteomeCentral under accession no. PXD063748 and in the Mass Spectrometry Interactive Virtual Environment (MassIVE) database under accession no. MSV000097826. Source data are provided with this paper.

## Code availability

Code used in the analysis is available at the following GitHub repository: https://github.com/ukaraoz/blodgett_omics_manuscript_analysis (https://doi.org/10.5281/zenodo.21045432).
