# Supplementary material for: Soil proteogenomics uncover microbial strategies for soil depth-specific carbon cycling under whole-soil warming conditions
Source: Nat Commun. 2026 Oct 13;17:10740. doi: 10.1038/s41467-026-78137-0 (PMC13661901; doi:10.1038/s41467-026-78137-0)
Supplement: Supplementary file 1 [file 41467_2026_78137_MOESM1_ESM.pdf]

nature portfolio

Corresponding author(s): Eoin L. Brodie

Last updated by author(s): Jun 28, 2026

Reporting Summary

Nature Portfolio wishes to improve the reproducibility of the work that we publish. This form provides structure for consistency and transparency in reporting. For further information on Nature Portfolio policies, see our Editorial Policies and the Editorial Policy Checklist.

Statistics

For all statistical analyses, confirm that the following items are present in the figure legend, table legend, main text, or Methods section.

n/a	Confirmed	
☐	☒	The exact sample size (*n*) for each experimental group/condition, given as a discrete number and unit of measurement
☐	☒	A statement on whether measurements were taken from distinct samples or whether the same sample was measured repeatedly
☐	☒	The statistical test(s) used AND whether they are one- or two-sided<br>*Only common tests should be described solely by name; describe more complex techniques in the Methods section.*
☐	☒	A description of all covariates tested
☐	☒	A description of any assumptions or corrections, such as tests of normality and adjustment for multiple comparisons
☐	☒	A full description of the statistical parameters including central tendency (e.g. means) or other basic estimates (e.g. regression coefficient) AND variation (e.g. standard deviation) or associated estimates of uncertainty (e.g. confidence intervals)
☐	☒	For null hypothesis testing, the test statistic (e.g. F, t, r) with confidence intervals, effect sizes, degrees of freedom and P value noted<br>*Give P values as exact values whenever suitable.*
☒	☐	For Bayesian analysis, information on the choice of priors and Markov chain Monte Carlo settings
☐	☒	For hierarchical and complex designs, identification of the appropriate level for tests and full reporting of outcomes
☒	☐	Estimates of effect sizes (e.g. Cohen's *d*, Pearson's *r*), indicating how they were calculated

Our web collection on statistics for biologists contains articles on many of the points above.

Software and code

Policy information about availability of computer code

Data collection	We didn't use any software for data collection.
Data analysis	metaSPAdes v3.15.3<br>Prodigal v2.6.3<br>USEARCH v8.1.1861_i86linux64<br>Bowtie2 v2.2.6<br>CoverM v0.7.0<br>MAFFT v7.427<br>FastTree v2.1<br>COCACOLA 1.1.0<br>MaxBin2.0<br>MetaBAT2<br>HMMER 3.2<br>DAS Tool v1.1<br>dRep v3.4.2<br>R v3.4.0. Specific R packages and statistical functions used in analysis are detailed in Methods.<br>Workflows describing the custom analysis code used in this study are available as described in the Code Availability Statement.

For manuscripts utilizing custom algorithms or software that are central to the research but not yet described in published literature, software must be made available to editors and reviewers. We strongly encourage code deposition in a community repository (e.g. GitHub). See the Nature Portfolio guidelines for submitting code & software for further information.

Data

Policy information about availability of data

All manuscripts must include a data availability statement. This statement should provide the following information, where applicable:

- Accession codes, unique identifiers, or web links for publicly available datasets
- A description of any restrictions on data availability
- For clinical datasets or third party data, please ensure that the statement adheres to our policy

Metagenomics sequencing are available under NCBI BioProject accession nos PRJNA1225762 and PRJEB39497, and JGI GOLD Study Gs0151586 (refer to Supplementary Table 2 for sample level details). Metagenome-assembled genomes and associated metadata are available on the Environmental System Science Data Infrastructure for a Virtual Ecosystem (ESS-DIVE) repository (Karaoz et al. doi: 10.15485/2588706). Raw metaproteomic data acquisitions from Mass Spectrometer are available in EMSL Science Central under EMSL project ID 50964 (https://sc-data.emsl.pnnl.gov/?projectId=50964). The data products from MS-GF + searches are also deposited in the Mass Spectrometry Interactive Virtual Environment (MassIVE) database under accession no. MSV000097826.
Code used in the analysis is available at the following GitHub repository: https://github.com/ukaraoz/blodgett_omics_manuscript_analysis.

Research involving human participants, their data, or biological material

Policy information about studies with human participants or human data. See also policy information about sex, gender (identity/presentation), and sexual orientation and race, ethnicity and racism.

Reporting on sex and gender	*Use the terms sex (biological attribute) and gender (shaped by social and cultural circumstances) carefully in order to avoid confusing both terms. Indicate if findings apply to only one sex or gender; describe whether sex and gender were considered in study design; whether sex and/or gender was determined based on self-reporting or assigned and methods used.*<br>*Provide in the source data disaggregated sex and gender data, where this information has been collected, and if consent has been obtained for sharing of individual-level data; provide overall numbers in this Reporting Summary. Please state if this information has not been collected.*<br>*Report sex- and gender-based analyses where performed, justify reasons for lack of sex- and gender-based analysis.*
Reporting on race, ethnicity, or other socially relevant groupings	*Please specify the socially constructed or socially relevant categorization variable(s) used in your manuscript and explain why they were used. Please note that such variables should not be used as proxies for other socially constructed/relevant variables (for example, race or ethnicity should not be used as a proxy for socioeconomic status).*<br>*Provide clear definitions of the relevant terms used, how they were provided (by the participants/respondents, the researchers, or third parties), and the method(s) used to classify people into the different categories (e.g. self-report, census or administrative data, social media data, etc.)*<br>*Please provide details about how you controlled for confounding variables in your analyses.*
Population characteristics	*Describe the covariate-relevant population characteristics of the human research participants (e.g. age, genotypic information, past and current diagnosis and treatment categories). If you filled out the behavioural & social sciences study design questions and have nothing to add here, write "See above."*
Recruitment	*Describe how participants were recruited. Outline any potential self-selection bias or other biases that may be present and how these are likely to impact results.*
Ethics oversight	*Identify the organization(s) that approved the study protocol.*

Note that full information on the approval of the study protocol must also be provided in the manuscript.

Field-specific reporting

Please select the one below that is the best fit for your research. If you are not sure, read the appropriate sections before making your selection.

☐ Life sciences ☐ Behavioural & social sciences ☒ Ecological, evolutionary & environmental sciences

For a reference copy of the document with all sections, see nature.com/documents/nr-reporting-summary-flat.pdf

Ecological, evolutionary & environmental sciences study design

All studies must disclose on these points even when the disclosure is negative.

Study description	The study site is located in the University of California Blodgett Experimental Forest in the Sierra Nevada foothills of California at 1370 m above sea level (120°39'40''W; 38°54'43''N) (Hicks Pries et al. 2017). The average yearly precipitation at the site is 1660 mm, with most falling as snow between November and April. The mean annual air temperature is approximately 12.5 °C. The site is in a thinned mixed conifer forest with dominant overstory species of ponderosa pine (Pinus ponderosa), sugar pine (Pinus lambertiana), incense cedar (Calocedrus decurrens), white fir (Abies concolor), and douglas fir (Pseudotsuga menziesii). The soil order at the site is classified as Alfisol of granitic origin with a developed O horizon as part of the Holland-Bighill complex.<br><br>The field warming experiment, described in detail in Hicks Pries et al and in its Supplementary Methods (Hicks Pries et al. 2017), started in October 2013 when the heaters were turned on. Shortly, the experimental design includes three pairs of heated plots and

	unheated control plots, each circular with a 3 m diameter and each pair of heated and control plots situated next to each other. The coordinates corresponding to each of the three paired plots are given in Supplementary Table 1. Plots within a pair are adjacent and located in the same stand conditions to minimize differences in overstory, slope, and soils, with the control plot providing the local reference for the heated one. The distances between the 3 paired experimental plots are 21m, 25m, and 34m. Each plot is circular, 3m in diameter. To minimize the effect of disturbance across different years, soil cores were taken between heating rods and away from temperature probes, maintaining the integrity of the heating system and the monitoring instrumentation, and avoiding repeated sampling in the same plot regions. Heating treatment followed the design of Hanson et al. (Hanson et al. 2017) with resistance heating cables (BriskHeat, Ohio, USA) that warm soil by 4°C above ambient temperature down to 1 m depth, while preserving the natural temperature gradient through the soil profile. Unheated cables were placed in the paired control plots using the same design. Soil temperature and soil volumetric water content were monitored continuously across the depth profile using temperature sensors (Omega 44005) and enviroSCAN (Sentek, Australia) probes. These measurements were used as input to a system that drives the power to the heaters in order to maintain the targeted 4°C warming in the paired heated plots (see Hicks Pries et al (Hicks Pries et al. 2017) and Soong et al. (Soong et al. 2021) for details).
Research sample	We collected soil cores from each plot using a 5-cm diameter AMS corer (AMS Inc. Hayward Falls, ID, USA) at six distinct time points: 0.5, 1.5, 2.5, 3.5, 4.5, and 7.5 years after the warming treatment began. The soil depth intervals spanned from surface to 90 cm deep. The exact intervals changed across sampling years due to field practicalities. Within each field season, the same depth intervals were used for all plots. Supplementary Table 1 lists the depth intervals for all the samples.<br>For each sample, we extracted DNA from 0.25-0.5 g of field-moist soil using DNeasy PowerSoil Kit (Qiagen, Germantown, MD, USA) for metagenomic sequencing. For 2014, 2015, and 2021 samples, metagenomic library preparation and DNA sequencing were performed at the Joint Genome Institute. Samples from 2016 and 2018 were sequenced at the UCSF Center for Advanced Technology as described by Dove et al. 2021.<br>Samples from soil cores collected after 4.5 years of warming from control and warming plots were selected for metaproteomics analysis (30 samples = 3 replicates x 2 (control/warmed) x 5 depth intervals). Soil protein samples were extracted, digested, purified and analyzed by liquid chromatography-tandem mass spectrometry (LC-MS/MS), as previously described by (Nicora et al. 2018).
Sampling strategy	Sample size was determined by the number of in-situ plots (paired control (n=3) and heated (n=3) plots) and the depth intervals. We collected soil samples across 6 time points (corresponding to 0.5, 1.5, 2.5, 3.5, 4.5, and 7.5 years after warming) from 3 paired plots. Across years, the depth profiles were uneven with depth intervals due to the field practicality of sampling deep soil profiles (Figure 1B). Soil cores were collected from each plot using a 5-cm diameter AMS corer (AMS Inc. Hayward Falls, ID, USA) and extracted the depth intervals (Supp. Table 1) across years that correspond to warming treatment lengths. Single-use sterilized polycarbonate core sleeves were used to protect the soil cores. Soil samples were homogenized in sterile bags and were immediately placed on dry ice and stored at -80°C DNA extraction. Overall, there were 114 total soil samples, 95 of which yielded a successful metagenome. The total sample size for the proteomics experiments was 30 samples, corresponding to 4.5 years after warming. A formal analysis of statistical power was not performed.
Data collection	For 2014, 2015, and 2021 samples, metagenomic sequencing were performed at the Joint Genome Institute. Samples from 2016 and 2018 were sequenced at the UCSF Center for Advanced. Metaproteomics data collection was performed at the Environmental Molecular Sciences Laboratory (EMSL) at Pacific Northwest National Laboratory (PNNL).
Timing and spatial scale	As noted in the sampling strategy, we collected samples from 6 time points (corresponding to 0.5, 1.5, 2.5, 3.5, 4.5, and 7.5 years after warming) from 3 paired plots and from depth intervals shown in Figure 1B. The time points across different years corresponded to peak microbial activity at the end of the rainy season in May/June of the corresponding years. Pairs of heated plots and unheated control plots are circular with a 3 m diameter. Sampling across different years was from separate subplot and up to a depth of 1 m, with depth intervals as specified in Figure 1B.
Data exclusions	No data was excluded. We attempted to extract DNA for metagenomics from all collected soil samples. Among 114 total soil samples, 95 yielded a successful metagenome. The total sample size for the proteomics experiments was 30 samples, corresponding to all collected samples from 4.5 years after warming.
Reproducibility	All metagenome and metaproteome measurements reported here could only be performed once per sampled year/depth/plot combinations. As we analyzed samples collected directly from the environment, it is not feasible for us to repeat the experiment under the same conditions.
Randomization	Our study has a block design contrasting controls with heating treatment. Paired field experimental plots were setup identically and were randomly assigned +4C heating or control (ambient) heating. For each sampling year, a random subplot was chosen for collecting soil cores from which the specified depth intervals were analyzed.
Blinding	Not applicable as we do not study human subjects.

Did the study involve field work? ☒ Yes ☐ No

Field work, collection and transport

Field conditions	Samples are collected at end end of the rainy season. The average yearly precipitation at the site is 1660 mm, with most falling as snow between November and April. The mean annual air temperature is approximately 12.5 °C.
Location	The study site is located in the University of California Blodgett Experimental Forest in the Sierra Nevada foothills of California at 1370 m above sea level (120°39'40''W; 38°54'43''N)
Access & import/export	Permissions granted to LBNL Belowground Biogeochemistry Scientific Focus Area (SFA) by the University of California Berkeley.
Disturbance	Soils that are destructively sampled were filled. No plant or animal life was disturbed during sampling.

Reporting for specific materials, systems and methods

We require information from authors about some types of materials, experimental systems and methods used in many studies. Here, indicate whether each material, system or method listed is relevant to your study. If you are not sure if a list item applies to your research, read the appropriate section before selecting a response.

Materials & experimental systems

n/a	Involved in the study
☒	☐ Antibodies
☒	☐ Eukaryotic cell lines
☒	☐ Palaeontology and archaeology
☒	☐ Animals and other organisms
☒	☐ Clinical data
☒	☐ Dual use research of concern
☒	☐ Plants

Methods

n/a	Involved in the study
☒	☐ ChIP-seq
☒	☐ Flow cytometry
☒	☐ MRI-based neuroimaging

Plants

Seed stocks	Not applicable
Novel plant genotypes	Not applicable
Authentication	Not applicable
