# Supplementary material for: Soil proteogenomics uncover microbial strategies for soil depth-specific carbon cycling under whole-soil warming conditions
Source: Nat Commun. 2026 Oct 13;17:10740. doi: 10.1038/s41467-026-78137-0 (PMC13661901; doi:10.1038/s41467-026-78137-0)
Supplement: Supplementary file 2 [file 41467_2026_78137_MOESM2_ESM.pdf]

Supplementary Figure 1

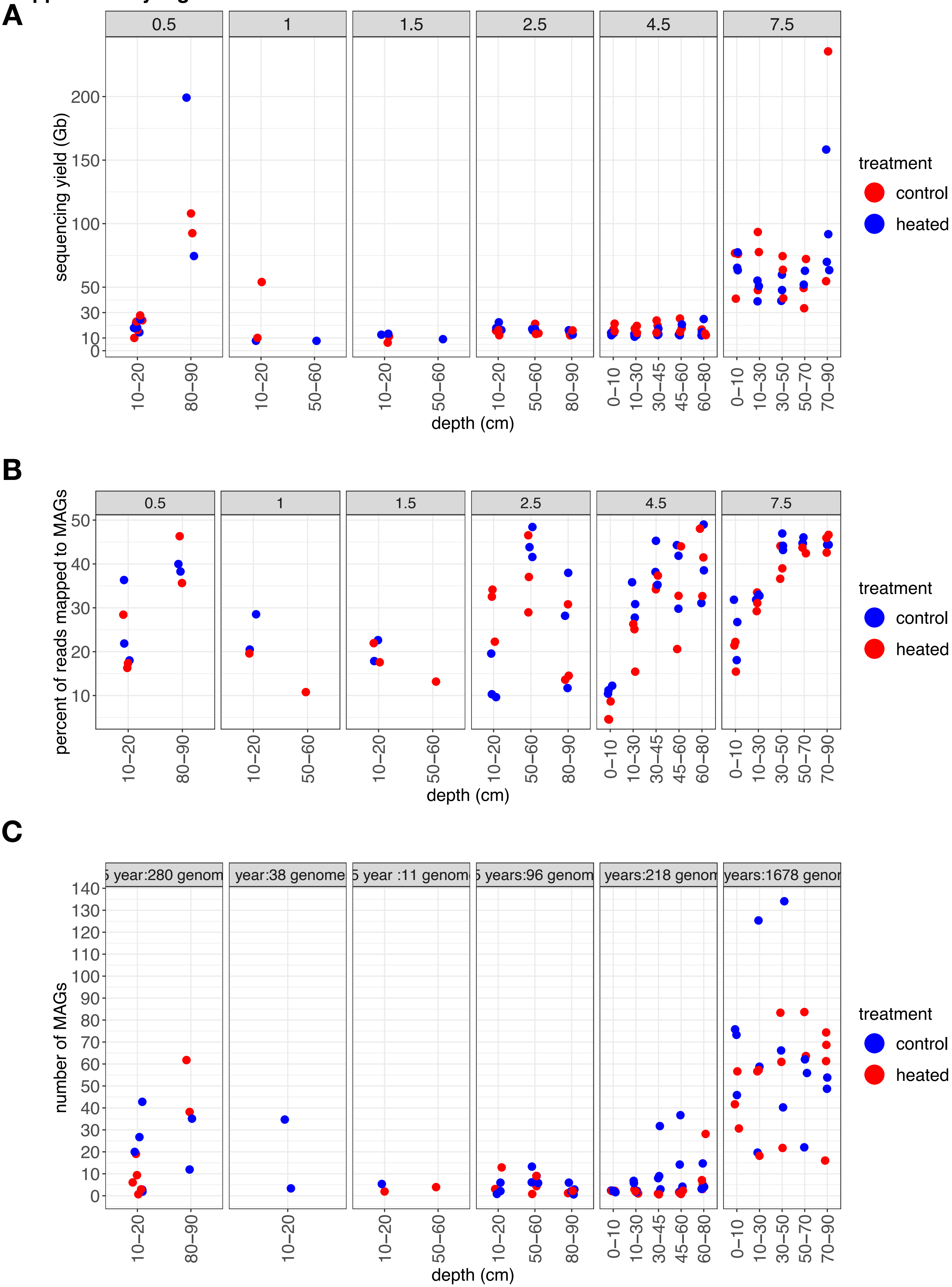

Supplementary Figure 1. Sample level metagenomic sequencing effort (A), read representativeness as percent mapped reads (B), and the resulting reconstructed genomes stratified by depth, year, and plot type. Source data are provided as a Source Data file.

Supplementary Figure 2

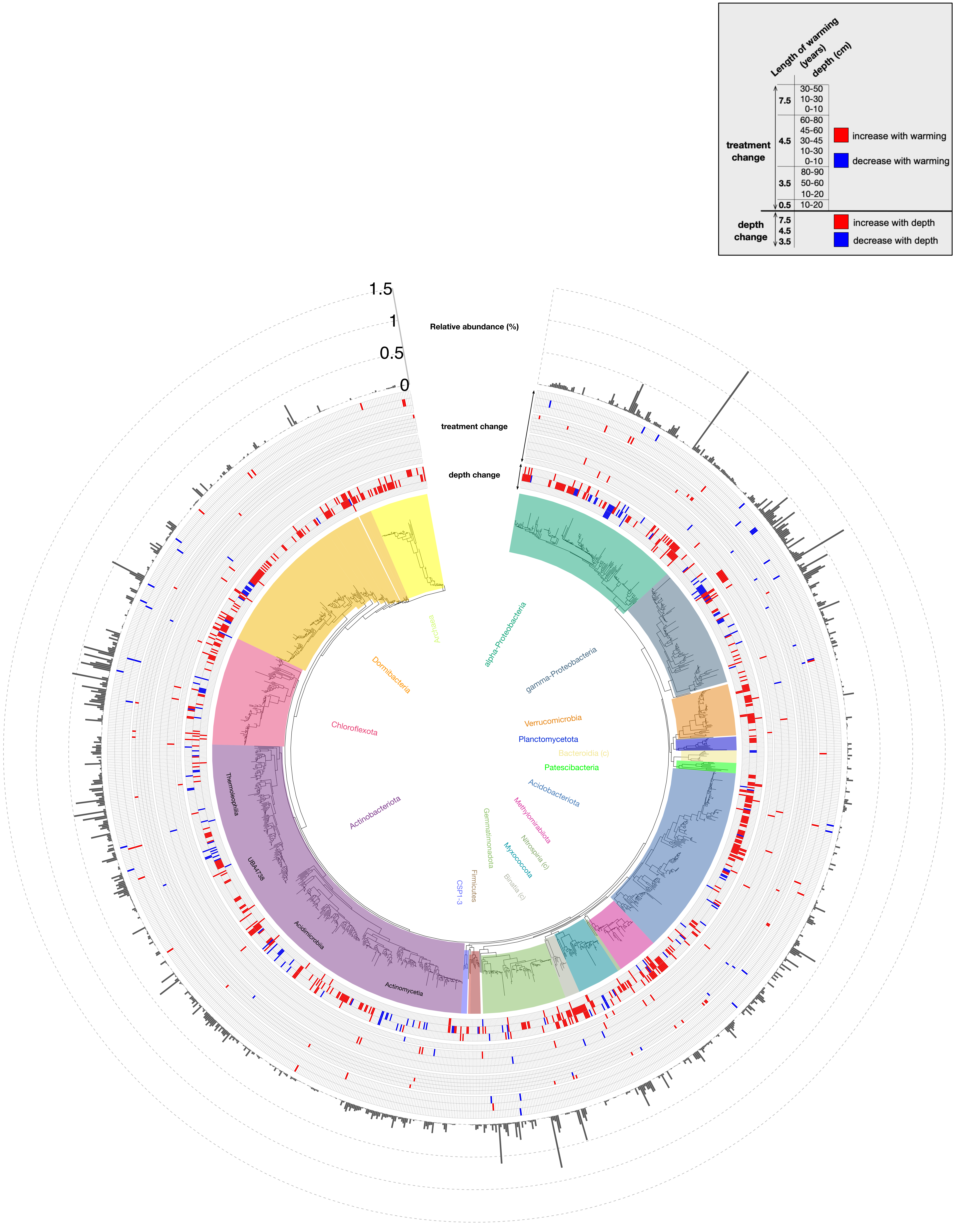

Supplementary Figure 2. Maximum likelihood phylogenetic tree of all high and medium quality genomes with mean abundance (control plots), depth and warming treatment response. Phylogenetic tree was constructed with a concatenated alignment of 15 co-located ribosomal proteins (L2, L3, L4, L5, L6, L14, L15, L16, L18, L22, L24, S3, S8, S17 and S19). Concentric rings from the tree shows if the genome's abundance was significantly different across depth and treatment for comparisons shown in the legend. The direction of significant response (increase/decrease with depth/warming treatment) is shown with red/blue color. The outermost ring shows the mean relative abundance across all control plots. The clades are colored based on the assigned GTDB taxonomy at phylum/class level.

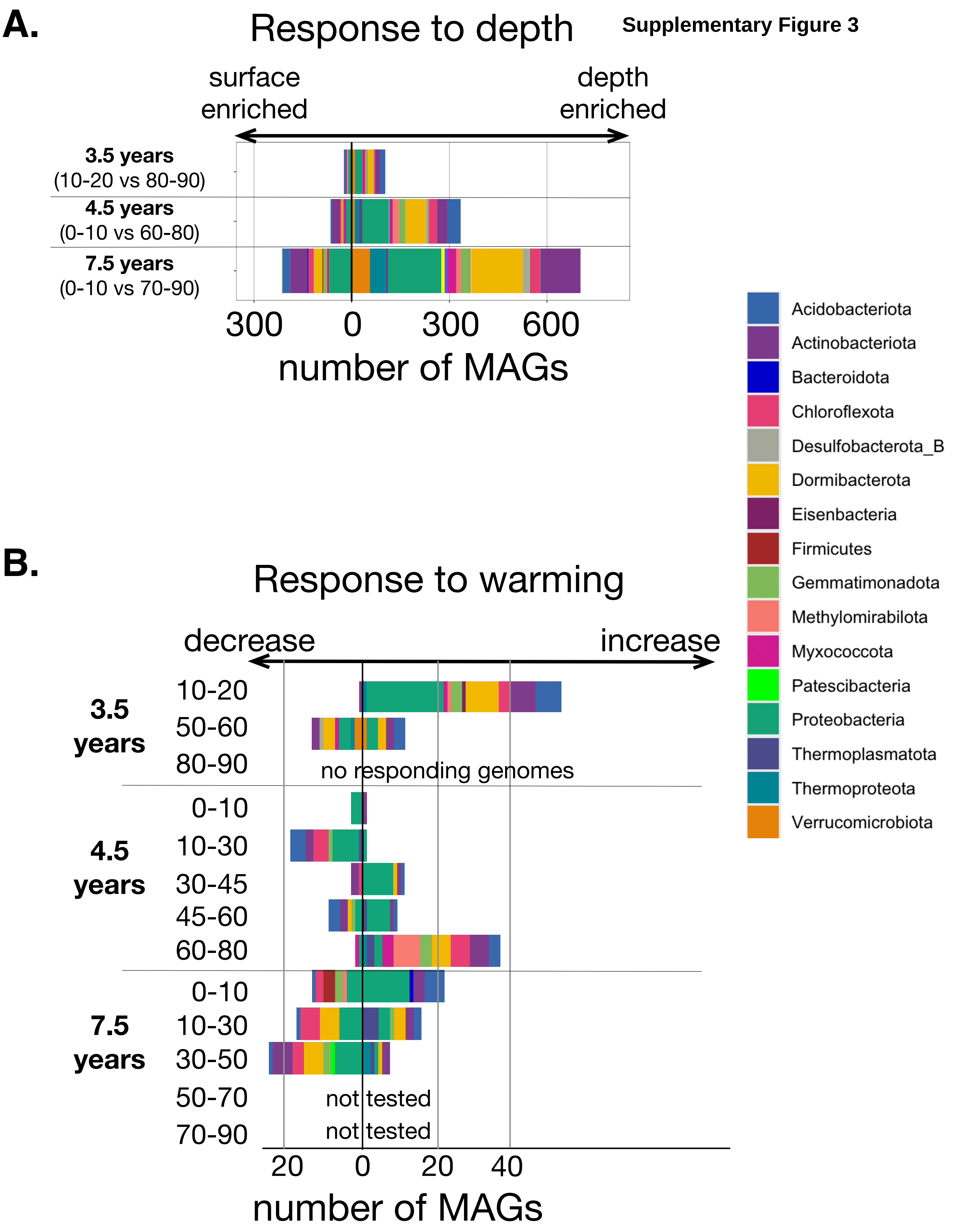

A.
Response to depth
Supplementary Figure 3
surface enriched
depth enriched
3.5 years
(10-20 vs 80-90)
4.5 years
(0-10 vs 60-80)
7.5 years
(0-10 vs 70-90)
300
0
300
600
number of MAGs
Acidobacteriota
Actinobacteriota
Bacteroidota
Chloroflexota
Desulfobacterota_B
Dormibacterota
Eisenbacteria
Firmicutes
Gemmatimonadota
Methylomirabilota
Myxococcota
Patescibacteria
Proteobacteria
Thermoplasmatota
Thermoproteota
Verrucomicrobiota
B.
Response to warming
decrease
increase
3.5 years
10-20
50-60
80-90
no responding genomes
4.5 years
0-10
10-30
30-45
45-60
60-80
7.5 years
0-10
10-30
30-50
50-70
not tested
70-90
not tested
20
0
20
40
number of MAGs

Supplementary Figure 3. Phyla level distributions of significantly enriched genomes by depth and warming treatment. The figure shows the number of MAGs and their phyla level distribution that underlie community level shifts reported in Fig 2 with depth (A) and warming treatment (B). Right/left of the zero line is for genomes significantly increasing/decreasing with depth or warming. (A) Distribution of significantly enriched MAGs at surface and depth where the comparison is between the shallowest and deepest depth interval from the sampling year. (B) Distribution of significantly enriched MAGs with warming. Source data are provided as a Source Data file.

Supplementary Figure 4

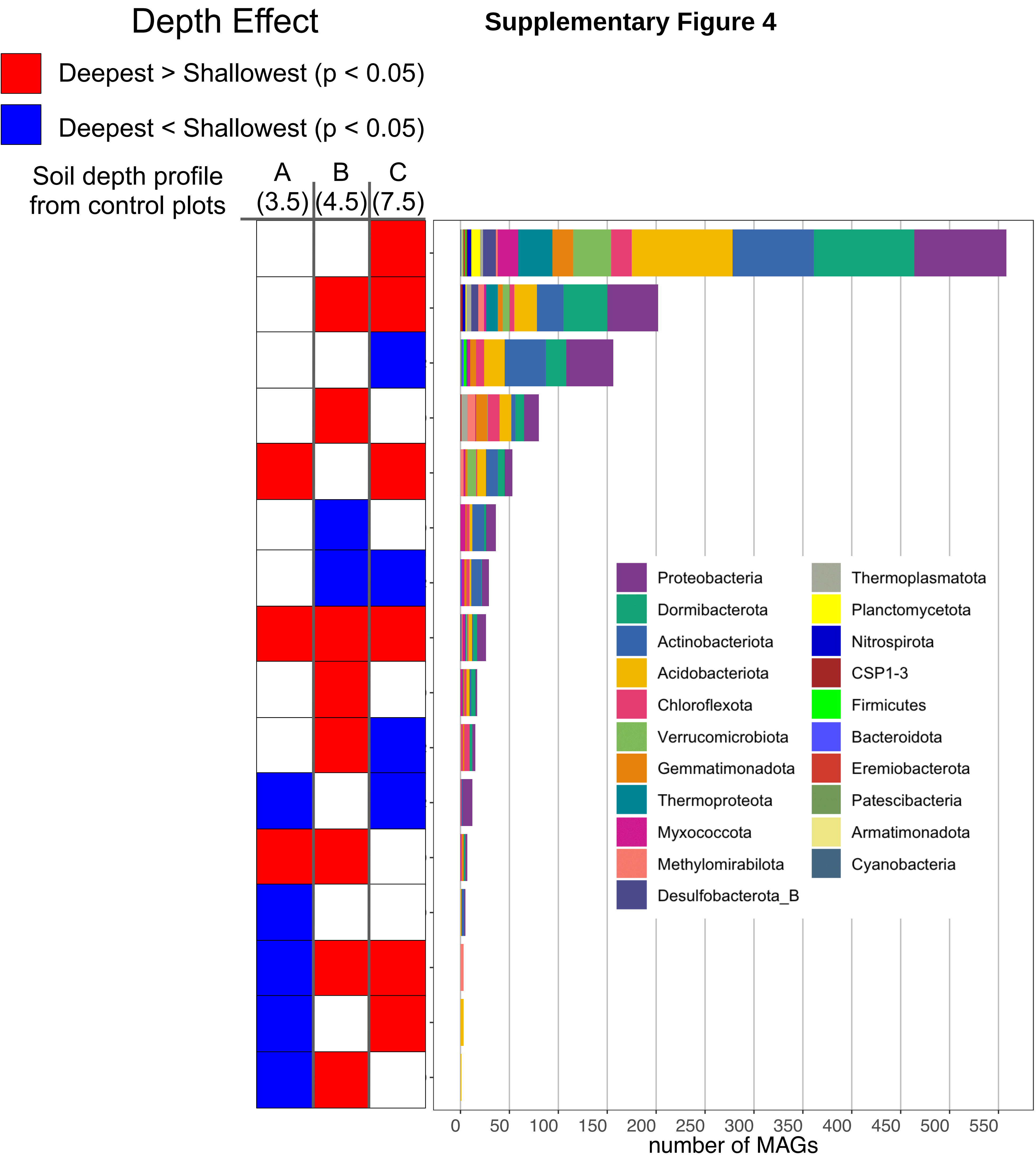

Supplementary Figure 4. MAG response to depth across years of warming treatment from control plots. The figure shows the richness (number of MAGs) and diversity (phylum association) of MAGs significantly differing across the depth profile across years broken down by the pattern of the response (red: increase with depth, blue: decrease with depth). Source data are provided as a Source Data file.

Supplementary Figure 5

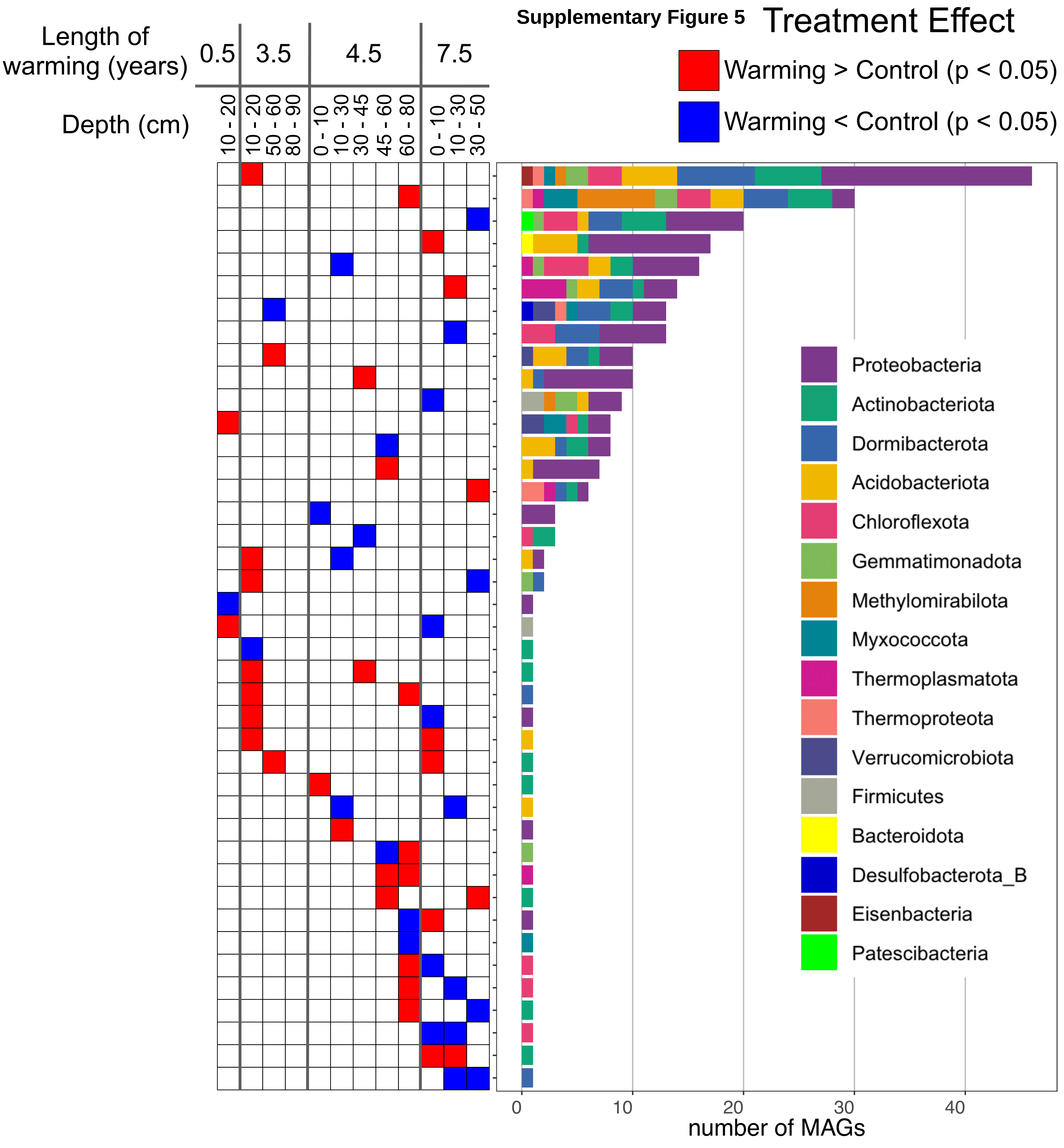

Supplementary Figure 5. MAG response to warming treatment across years of warming treatment. The figure shows the richness (number of MAGs) and diversity (phylum association) of MAGs significantly differing between control and warming plots across years broken down by the pattern of the response (red: increase with warming, blue: decrease with warming) across years. Source data are provided as a Source Data file.

Supplementary Figure 6

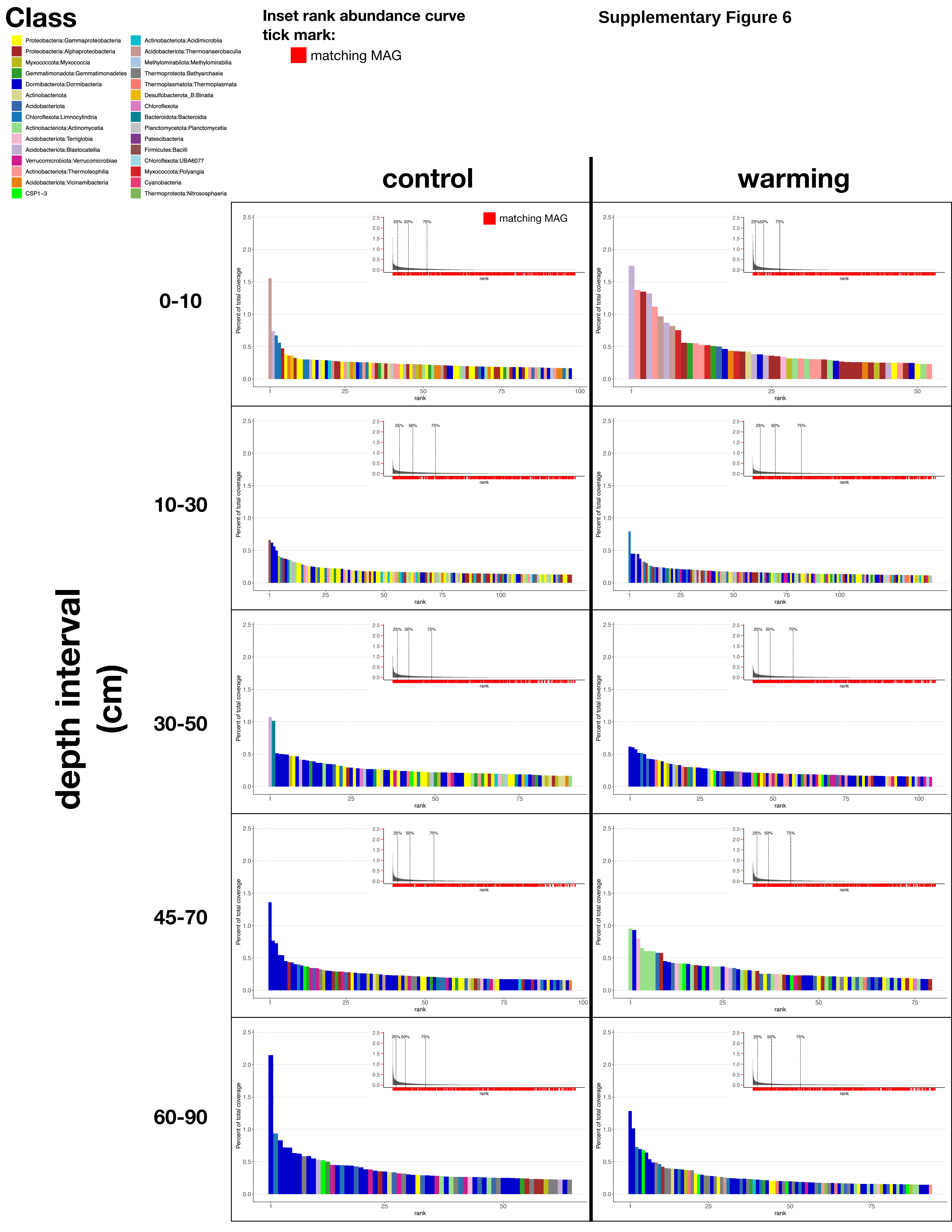

Supplementary Figure 6. Rank abundance curves of rpsC species groups and their binning status. Each bar corresponds to a species group colored by the class level lineage. The main rank abundance curves show the ranks up to 25% percentile, the inset shows the full distribution together with the binning status (red tick marks underneath x-axis) corresponding to the species group. Source data are provided as a Source Data file.

Supplementary Figure 7

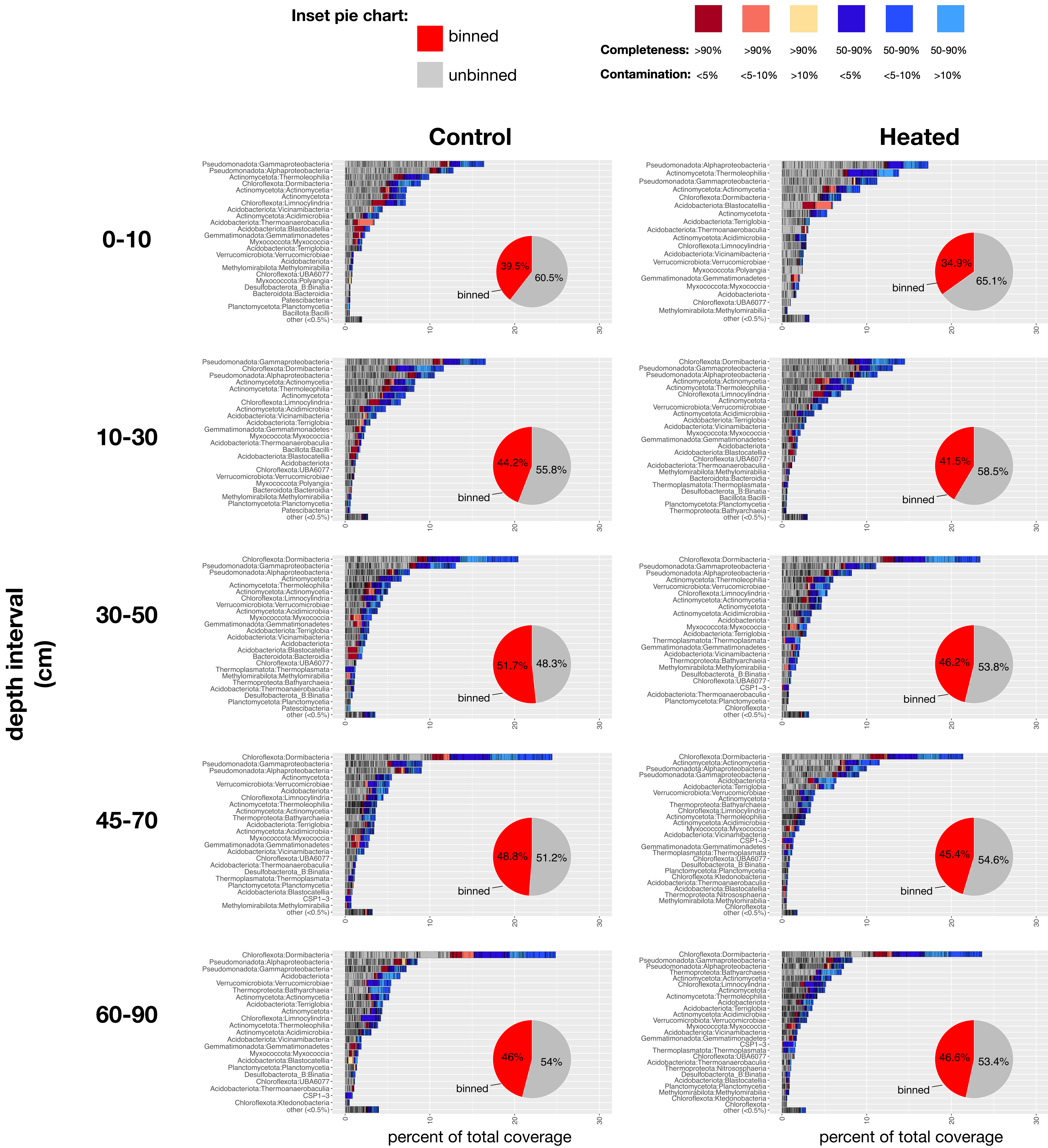

Supplementary Figure 7. Class level distribution of genome recovery for species groups and overall representativeness of the bins. Each bar shows the species groups associated with a specific class, colored by binning status and bin quality. Inset pie charts shows the breakdown of species groups associated with a bin (red) based on coverage weighted counts. Source data are provided as a Source Data file.

Supplementary Figure 8

Peptide Identification Strategy

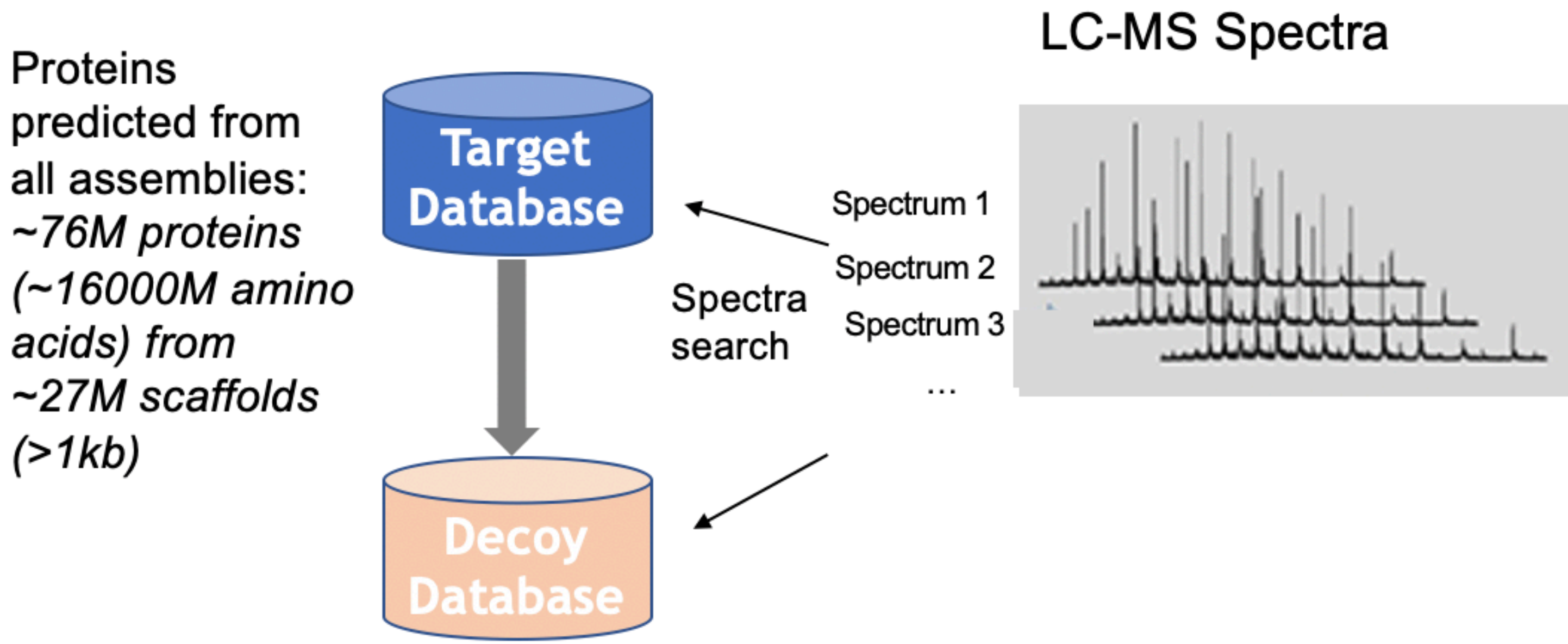

Estimate False Discovery Rate

**Spectrum**	**Score**	
	Target	Decoy
S1	0.65	0.22
S2	0.8	0.1
S3	0.1	0.95
...		

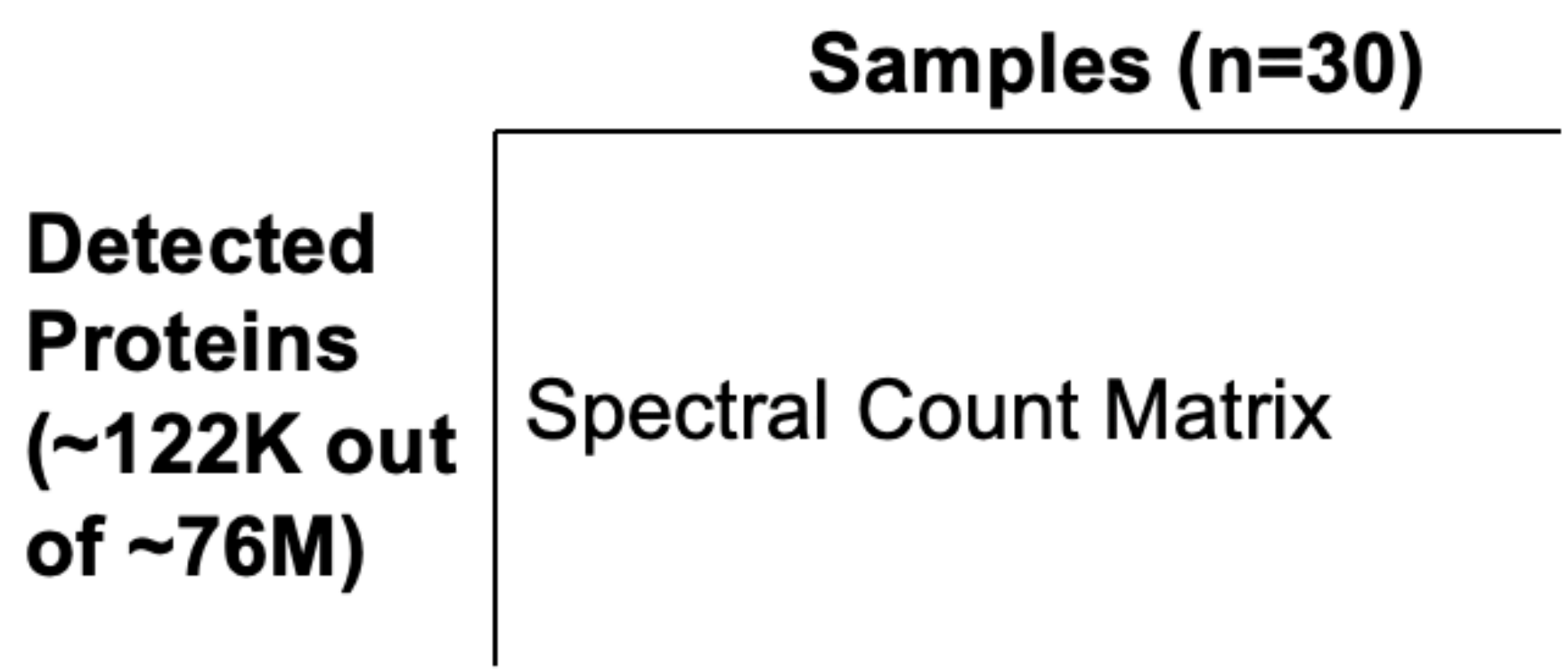

Supplementary Figure 8. Metagenome-centric protein identification workflow.
