# Supplementary material for: Soil proteogenomics uncover microbial strategies for soil depth-specific carbon cycling under whole-soil warming conditions
Source: Nat Commun. 2026 Oct 13;17:10740. doi: 10.1038/s41467-026-78137-0 (PMC13661901; doi:10.1038/s41467-026-78137-0)
Supplement: Supplementary file 3 [file 41467_2026_78137_MOESM5_ESM.pdf]

nature portfolio

Peer Review File

Soil proteogenomics uncover microbial strategies for soil depth-specific carbon cycling under whole-soil warming conditions

Corresponding Author: Dr Eoin Brodie

This file contains all reviewer reports in order by version, followed by all author rebuttals in order by version.

Version 0:

Reviewer comments:

Reviewer #1

(Remarks to the Author)
Dear Editor,
Please find below my comments on the manuscript entitled "Whole Soil Proteogenomics Uncover Hidden Microbial Strategies for Carbon Cycling Across the Soil Profile Under Deep Soil Warming" written by Karaoz et al. The manuscript examines how soil microbes at different depths regulate carbon cycling under long-term warming. The authors propose that depth-structured microbial traits, together with local environmental conditions, shape how soil respiration responds to warming. They address three components of this framework: variation in microbial communities and traits across depth, the response of these traits to long-term warming, and the metabolic pathways that underlie these depth-specific and warming-driven processes.

The study was conducted at the UC Blodgett Experimental Forest in the Sierra Nevada foothills of California, where soils have been warmed by +4 °C since 2014. Over 7.5 years, the authors generated 101 metagenomes from 95 soil samples (yielding 2,321 dereplicated MAGs) and produced 30 metaproteomes that provide molecular evidence of in situ functional potential. These data show that depth is the dominant factor shaping microbial communities, while warming produces smaller but meaningful shifts in substrate use, C1 metabolism, nitrogen transformations, and stress responses. The study represents a substantial effort and has strong potential to advance understanding of how ecosystems respond to warming. Nevertheless, several methodological and presentation-related points should be addressed to strengthen the manuscript.

1. Physiological interpretation of warming responses and the need for biomass constraints
Soil warming increases the energetic costs required for microbes to maintain cellular viability, including membrane stability, protein repair, and replacement of thermally damaged enzymes. These elevated maintenance demands often shift resource allocation away from growth and toward survival. At the ecosystem scale, this is typically reflected in increased CO_2 production, but respiration alone is insufficient to interpret microbial responses. Without information on microbial biomass, it is challenging to determine whether higher CO_2 fluxes result from increased per-cell activity or shifts in population size or composition.
Although direct measurement of microbial biomass may be challenging in this system, qPCR or ddPCR quantification of major microbial groups and relevant functional genes would strengthen the interpretation of CO_2 flux patterns and help constrain changes in microbial abundance. These measurements would also enable more informed inferences about carbon use efficiency, a key determinant of soil carbon stability under warming conditions.

2. Nutrient availability and the need for soil chemical characterization
Meeting the increased energy demands imposed by global warming requires a greater acquisition of carbon, nitrogen, and phosphorus from the environment. The availability of these nutrients and the resulting soil C:N:P ratios directly influence which metabolic pathways and extracellular enzymes are up- or down-regulated. For this reason, including measurements of soil chemical properties (total carbon, total nitrogen, TOC, NH_4^+, NO_x^-, and, ideally, phosphorus fractions) would provide essential context for interpreting changes in genomic and proteomic trait distributions.

This is particularly relevant because the authors observe declines in DNRA capacity alongside increases in ureases, nitrous oxide reductases, and nitric oxide reductases. Such patterns may indicate a shift toward nitrogen removal processes

typically associated with low soil C:N ratios and relative carbon limitation. Without soil nutrient data, however, it is difficult to confidently link the observed functional patterns to underlying biogeochemical conditions. Soil nutrient characterization would therefore substantially strengthen the ecological interpretation of the proteogenomic results.

3. Interpretation based on gene and protein presence and the need to address relic DNA and mineral-bound proteins
The manuscript interprets ecosystem responses based largely on the abundance and presence of genes and proteins detected in metagenomic and metaproteomic datasets. While this is broadly appropriate, clearer methodological justification is required because environmental DNA and proteins do not always reflect living, active microbial cells.

Environmental DNA may contain up to 80% relic DNA from dead or lysed cells. Its presence can bias community composition estimates and inflate apparent functional potential. The authors should clarify the DNA extraction procedure, whether relic DNA removal steps were used, and how relic DNA may influence depth- or warming-specific signals. Given the importance of this dataset, explicitly noting this limitation in both the Methods and Discussion sections would strengthen the conclusions.

Similarly, extracellular proteins can become tightly associated with minerals, remain extractable in the laboratory, but not represent biologically available or actively expressed proteins in situ. A brief discussion of how mineral-associated proteins may influence proteomic interpretation would further increase methodological transparency.

4. Data representation and figure clarity
Several figures are visually crowded and difficult to interpret. In Figure 2B, the small inset showing MAG abundances is dense and unconventional, requiring disproportionate effort to decipher. A similar issue is also observed in Figure 2C. In Figure 3, the heatmaps emphasize early-year changes but give the impression of a limited long-term effect, which may unintentionally contradict the authors' narrative.

The manuscript would benefit from alternative visualizations such as simplified trait groupings, clearer depth-stratified trends, separate panels for complex insets, or temporal line plots. These revisions would reduce visual clutter and more effectively highlight the major findings.

Reviewer #2

(Remarks to the Author)
The authors have studied the effect of soil heating over the period of 7.5 years on microbial communities and their activities across soil depth, which as far as we know has been little explored. To comprehensively assess these changes, authors used metagenomics and metaproteomics. Although, the authors show differential influence of increased temperature at different soil depths reflected in the shifts of metagenome, that are further confirmed by proteomics, the main finding is that this effect is only marginal compared to the effect of soil depth itself. Furthermore, the importance of soil depth in shaping microbial community composition has been previously shown, thus it would not be unexpected that due to community variation, soil depth would entail contrasting microbial metabolism and traits. In addition, we have several concerns regarding the study.
a) Sampling strategy is inconsistent throughout the duration of the experiment. This is illustrated in Fig 1B as in six sampling points only two of them are the same. These inconsistencies later translate to results and discussion that are harder to follow.
b) We are concerned about the vicinity of the plots, and whether the samples could be considered truly biologically independent at this scale (as shown in Fig. 1A). Moreover, several cores are sampled within the plot which opens a question whether the disturbance could have any effects.
c) The authors would like to challenge the glucose-centric model and present a convergence of microbial metabolism on LMW and C1 with increasing depth. However, the experimental set-up and location is very specific and its results cannot serve as a basis of a general model. The experiment has been running in a temperate conifer forest mostly dominated by species from Pinaceae family. Therefore, low soil pH would dictate the composition and functon(including enzymatic activities) of the microbes. Furthermore, the interaction with ectomycorrhizal fungi, abundant in mature forests dominated by species of Pinaceae family, would further drive the selection of other microbial species. Hence, this specific case is not representative of other ecosystems.
d) Figure 3. and Tab. 1 are hard to navigate. This is exacerbated by the inconsistencies in the experimental design.
e) A strong emphasis is placed on discussion of C and N metabolism; yet, a basic analysis of soil is missing such as C, N, P content and soil pH.
f) Moreover discussion could expand on how the metabolism of the analysed microbes interconnects with other major organisms present in the soil – plants and fungi.
L27 There are studies that have shown the effect of soil depth on microbial communities (e.g. https://doi.org/10.1128/AEM.02673-20)
L27 The link with warming effect does not seem smooth and gives the impression being an add-on study.
The manuscript contains many unexplained abbreviations both in the text (L55, L58, L65, L84, L85, L91) and in the Tab. 1

Reviewer #3

(Remarks to the Author)
The paper describes a field-based research project involving deep soil warming over several years. The soil was sampled at

multiple depths across years, and the soil microbial community was analyzed with a combination of metagenomics and meta-proteomics. The analyses show functionally distinct communities in the surface soils vs. the deeper horizons, as well as functional shifts due to prolonged warming. This paper is novel and important, and will be of great interest to the readership of Nature Communications. The paper is well-written, and most of my suggested revisions are minor and have to do with the figures and associated captions.
Specific comments:

Line 209 speaks to current earth system models, but cites only papers that are a decade old. There have been advances made in that timeframe to account for soil microbial traits, but still deep soil microbiomes are not considered. The papers cited are excellent, and the point is valid, but perhaps more nuance and more recent citations would help.

Figure 1 caption does not adequately indicate the importance of the colors in part B, and connect those colors to the colored soil cores indicated in part A. Along those lines, I think I figured out that the color coding of Figure 1 carries through the part A of Figure 2, which is good but should be given its own legend of some sort, but then part C uses a completely different shading with depth with the tiniest legend I have ever seen. I think part C should also use the shading used in Figure 1 part A, with different color by year and lighter colors with depth, rather than the darker with depth being used now. That consistency would help simplify.

Figure 3 – are each of these comparisons indicated with color for significant changes? I do think I like the use of presence/absence of change vs. heat map here, as it is easy to understand.
In the methods, the first sentence of the section “Soil sampling, DNA extraction and metagenome sequencing” was unclear and should be clarified. Also in this section, there is no explanation of the variable depths and variable binning of depth increments in different years, and the approach to the comparisons of data given differences in sampling between years. This justification and explanation is necessary.

Reviewer #4

(Remarks to the Author)
I co-reviewed this manuscript with one of the reviewers who provided the listed reports. This is part of the Nature Communications initiative to facilitate training in peer review and to provide appropriate recognition for Early Career Researchers who co-review manuscripts.

Open Access This Peer Review File is licensed under a Creative Commons Attribution 4.0 International License, which permits use, sharing, adaptation, distribution and reproduction in any medium or format, as long as you give appropriate credit to the original author(s) and the source, provide a link to the Creative Commons license, and indicate if changes were made.
In cases where reviewers are anonymous, credit should be given to 'Anonymous Referee' and the source.
The images or other third party material in this Peer Review File are included in the article’s Creative Commons license, unless indicated otherwise in a credit line to the material. If material is not included in the article’s Creative Commons license and your intended use is not permitted by statutory regulation or exceeds the permitted use, you will need to obtain permission directly from the copyright holder.
To view a copy of this license, visit https://creativecommons.org/licenses/by/4.0/

Response to referees: NCOMMS-25-90717-T: Karaoz et al. ***Whole-Soil Proteogenomics Uncover Hidden Microbial Strategies for Carbon Cycling Across the Soil Profile Under Deep Soil Warming***

REVIEWER 1

Comment 1.1: Physiological interpretation of warming responses and the need for biomass constraints. Soil warming increases the energetic costs required for microbes to maintain cellular viability, including membrane stability, protein repair, and replacement of thermally damaged enzymes. These elevated maintenance demands often shift resource allocation away from growth and toward survival. At the ecosystem scale, this is typically reflected in increased CO_2 production, but respiration alone is insufficient to interpret microbial responses. Without information on microbial biomass, it is challenging to determine whether higher CO_2 fluxes result from increased per-cell activity or shifts in population size or composition.

Although direct measurement of microbial biomass may be challenging in this system, qPCR or ddPCR quantification of major microbial groups and relevant functional genes would strengthen the interpretation of CO_2 flux patterns and help constrain changes in microbial abundance. These measurements would also enable more informed inferences about carbon use efficiency, a key determinant of soil carbon stability under warming conditions.

Response 1.1: We agree that robust physiological interpretation of warming responses will require taking into account biomass variation as a function of depth and warming treatment. Actually, in this field warming experiment, we measured the response of microbial biomass to warming and have previously shown that the response varies between the topsoil and subsoil, with some dependency on the measurement method used (Zosso et al. 2021). After 4.5-year of whole-soil warming, bulk measurements of Microbial Biomass Carbon (MBC) showed no significant change with warming at any soil depth (Zosso et al. 2021). However, targeted molecular analyses proved more sensitive at capturing the treatment effect (Zosso et al. 2021). Using Phospholipid Fatty Acid (PLFA) concentrations (considered to be a more precise proxy for living microbial biomass), we found that microbial biomass decreased by 28% in the subsoil (below 30 cm) but remained unaffected in the topsoil (Zosso et al. 2021). Similarly, concentrations of branched glycerol dialkyl glycerol tetraethers (brGDGTs), which could be considered indicators of microbial necromass, were also lower in warmed subsoils between 50 and 90 cm (Zosso et al. 2021). This pronounced decrease in subsoil microbial biomass is likely driven by carbon limitation; the warming treatment reduced fine root mass (Ofiti et al. 2021) and depleted unprotected soil organic carbon (Soong et al. 2021), depriving subsoil microorganisms of easily accessible organic matter (Zosso et al. 2021).

As the reviewer suggests biomass changes of specific microbial strains or functional groups could be achieved through qPCR or ddPCR assays, and our database of MAGs provides a critical resource for strain/functional group-specific primer design. This was beyond the scope of our

current study but we have expanded the discussion to point out the biomass trends and this future analytical possibility, reproduced below:

> "In this field warming experiment, we previously measured the response of microbial biomass to warming and have previously shown that the response varies between the topsoil and subsoil, with some dependency on the measurement method used (Zosso et al. 2021). After 4.5-year of whole-soil warming, bulk measurements of Microbial Biomass Carbon (MBC) showed no significant change with warming at any soil depth (Zosso et al. 2021). However, targeted molecular analyses proved more sensitive at capturing the treatment effect (Zosso et al. 2021). Using Phospholipid Fatty Acid (PLFA) concentrations, a more precise proxy for living microbial biomass, we found that microbial biomass decreased by 28% in the subsoil (below 30 cm) but remained unaffected in the topsoil (Zosso et al. 2021). Similarly, concentrations of branched glycerol dialkyl glycerol tetraethers (brGDGTs), which could be considered indicators of microbial necromass, were also lower in warmed subsoils between 50 and 90 cm (Zosso et al. 2021). This pronounced decrease in subsoil microbial biomass is likely driven by carbon limitation; the warming treatment reduced fine root mass (Ofiti et al. 2021) and depleted unprotected soil organic carbon (Soong et al. 2021), depriving subsoil microorganisms of easily accessible organic matter (Zosso et al. 2021). Our genome-resolved data provide opportunities for characterization of biomass changes of specific microbial strains or functional groups we report here through qPCR or ddPCR assays by allowing precise, custom primer design targeting uncultured microorganisms. Such measurements would strengthen the interpretation of CO_2 flux patterns and carbon use efficiency, a crucial factor controlling soil carbon retention under warming conditions (Tao et al. 2023; Tian et al. 2023; Zhang et al. 2024)."

Comment 1.2: Nutrient availability and the need for soil chemical characterization. Meeting the increased energy demands imposed by global warming requires a greater acquisition of carbon, nitrogen, and phosphorus from the environment. The availability of these nutrients and the resulting soil C:N:P ratios directly influence which metabolic pathways and extracellular enzymes are up- or down-regulated. For this reason, including measurements of soil chemical properties (total carbon, total nitrogen, TOC, NH_4^+, NO_x^-, and, ideally, phosphorus fractions) would provide essential context for interpreting changes in genomic and proteomic trait distributions.
This is particularly relevant because the authors observe declines in DNRA capacity alongside increases in ureases, nitrous oxide reductases, and nitric oxide reductases. Such patterns may indicate a shift toward nitrogen removal processes typically associated with low soil C:N ratios and relative carbon limitation. Without soil nutrient data, however, it is difficult to confidently link the observed functional patterns to underlying biogeochemical conditions. Soil nutrient characterization would therefore substantially strengthen the ecological interpretation of the proteogenomic results.

Response 1.2: We agree that providing context on soil chemical properties is essential for interpreting the functional patterns observed in our results. To address your point regarding

shifting C:N ratios, we report previously quantified and published total soil organic carbon (SOC) and total nitrogen concentrations across the 0–90 cm soil profile. While SOC and total nitrogen concentrations were significantly lower in the warmed subsoils (below 20 cm), the soil C:N ratios did not significantly differ between the control and warmed plots at any soil depth (Ofiti et al. 2021; Soong et al. 2021). Therefore, the observed functional shifts in nitrogen cycling do not appear to be driven by a warming-induced shift toward lower bulk soil C:N ratios.
Regarding inorganic nitrogen (NH_4^+ and NO_x^-), we measured these pools but found that inorganic nitrogen concentrations in the soil pore water were often below the detection limit (0.5 mg N/liter) (Soong et al. 2021). Nearly all of the dissolved nitrogen was present in organic forms, which indicates a very high demand for inorganic nitrogen in this system (Soong et al. 2021). Furthermore, while warming significantly increased dissolved organic carbon (DOC) concentrations in the pore water, it did not have a significant effect on dissolved nitrogen concentrations (Soong et al. 2021).
However, your hypothesis regarding relative carbon limitation is well supported by our published site data. Heterotrophic microorganisms in these Mediterranean forest soils are generally considered carbon limited (Dove et al. 2021), and this limitation increases with depth (Alves et al. 2021). Sustained warming likely exacerbated this carbon limitation over time, as evidenced by a pronounced depletion of easily accessible, unprotected free particulate organic matter (fPOM) in the warmed subsoils (Soong et al. 2021; Dove et al. 2021).
We updated the manuscript to explicitly integrate these published soil nutrient data (total C, total N, C:N ratios, and inorganic N dynamics) into our discussion, to more directly link the observed genomic and functional patterns to the underlying carbon limitation and biogeochemical conditions of the soil.

> "Our results demonstrate that warming affected inorganic nitrogen dynamics traits. These patterns may suggest a transition toward N‑removal pathways that usually occur under low soil C:N conditions and when carbon is relatively limiting. Previously, we showed that while SOC and total nitrogen concentrations were significantly lower in the warmed subsoils (below 20 cm), the soil C:N ratios did not significantly differ between the control and warmed plots at any soil depth (Ofiti et al. 2021; Soong et al. 2021). Therefore, the observed functional shifts in nitrogen cycling do not appear to be driven by a warming-induced shift toward lower bulk soil C:N ratios. On the other hand, our previous data well supports relative carbon limitation as a driver of increased microbial N-removal processes under warming. Mediterranean forest soils are generally considered carbon limited (Dove et al. 2021), and this limitation increases with depth (Alves et al. 2021). During our experiment, sustained warming likely exacerbated this carbon limitation over time, as evidenced by a pronounced depletion of easily accessible, unprotected free particulate organic matter (fPOM) in the warmed subsoils (Soong et al. 2021; Dove et al. 2021)."

Comment 1.3: Interpretation based on gene and protein presence and the need to address relic DNA and mineral-bound proteins. The manuscript interprets ecosystem responses based largely

on the abundance and presence of genes and proteins detected in metagenomic and metaproteomic datasets. While this is broadly appropriate, clearer methodological justification is required because environmental DNA and proteins do not always reflect living, active microbial cells.

Environmental DNA may contain up to 80% relic DNA from dead or lysed cells. Its presence can bias community composition estimates and inflate apparent functional potential. The authors should clarify the DNA extraction procedure, whether relic DNA removal steps were used, and how relic DNA may influence depth- or warming-specific signals. Given the importance of this dataset, explicitly noting this limitation in both the Methods and Discussion sections would strengthen the conclusions.

Similarly, extracellular proteins can become tightly associated with minerals, remain extractable in the laboratory, but not represent biologically available or actively expressed proteins in situ. A brief discussion of how mineral-associated proteins may influence proteomic interpretation would further increase methodological transparency.

Response 1.3: We acknowledge that interference from extracellular/relic DNA for metagenomics and extraction efficiency for metaproteomics are limitations of soil proteogenomics techniques. We agree that the former would inflate apparent functional potential, while the latter is expected to reduce detection sensitivity. However, for genome-resolved metagenomics, we expect that fragmented, damaged, low-abundance sequences (like relic DNA) fail to form MAGs. While we have no direct evidence from our study, nor do we know of direct empirical evidence, fragmented DNA has previously been linked to poor assembly outcome (Royalty and Steen 2019). Our expectation is that relic DNA results in assembly errors (deBruijn graph breakage) that reduce the recovery of quality MAGs. Regardless, the reviewer points out some limitations of the metaproteogenomics approach that are pretty much universal for soil studies. We now describe these limitations in the methods as follows:

> "Interference from extracellular/relic DNA for metagenomics and extraction efficiency for metaproteomics are limitations of soil proteogenomics techniques. Our genome, rather than read-centric, analysis workflow remedies the effect of extracellular DNA by requiring the functional traits to be detected within genomes rather than within individual reads recruited to a database of sequences. While we have no direct evidence from our study, nor do we know of any direct empirical evidence, fragmented DNA has previously been linked to poor assembly outcome (Royalty and Steen 2019). Our expectation is that relic DNA results in assembly errors (de Bruijn graph breakage) that reduce the recovery of quality MAGs. While extracellular DNA might have inflated apparent functional potential, extraction efficiency for proteomics limits detection sensitivity.
>
> In soils, a substantial fraction of proteins becomes tightly associated with clay minerals, Fe/Al oxides, and mineral-associated organic matter, and extraction tests with radiolabeled proteins indicate that even strong extractants fail to recover the full mineral-bound pool (Greenfield et al. 2018; Yu et al. 2013). This suggests that proteins

from non-living cells (relic proteins) are less likely to contribute to our proteomic observations. Furthermore, during extraction we used an amino-acid–blocking soil protein extraction protocol (Nicora et al. 2018), which reduces sorption of cellular proteins to minerals (Nicora et al. 2013). Therefore, our metaproteomic data should be interpreted as representing the cellular soluble, and weakly sorbed subset of the soil proteome. With this in mind, it is theoretically possible that some of the observed protein expression differences could be attributed to variable protein sorption due to mineralogical differences through the soil profile."

"For soil protein extraction, we employed an amino-acid–blocking soil protein extraction protocol to minimize sorption losses to minerals. Soils were pre-incubated with a mixture of free amino acids to saturate mineral adsorption sites before in situ detergent lysis and proteome recovery as previously described in (Nicora et al. 2018)."

Comment 1.4: Data representation and figure clarity. Several figures are visually crowded and difficult to interpret. In Figure 2B, the small inset showing MAG abundances is dense and unconventional, requiring disproportionate effort to decipher. A similar issue is also observed in Figure 2C. In Figure 3, the heatmaps emphasize early-year changes but give the impression of a limited long-term effect, which may unintentionally contradict the authors' narrative.

The manuscript would benefit from alternative visualizations such as simplified trait groupings, clearer depth-stratified trends, separate panels for complex insets, or temporal line plots. These revisions would reduce visual clutter and more effectively highlight the major findings.

Response 1.4: We thank the reviewer for the suggested improvements. We updated Figure 2B and 2C, moving the insets showing taxonomic distributions of MAGs for depth (2B) and for warming (2C) to a new Supplementary Figure (Supp.Figure 3 in the revised manuscript).
We want to keep Figure 3 as is, organized by increasing depths and the years nested within the depths. This choice was intentional as the short vs. long-term effects should be interpreted in the context of depth intervals, and due to the need to consolidate the irregular sampling of depth intervals across years. For the trait groupings, the figure is already a simplified summary of traits after keeping depth/warming length-specific signatures, with the full list provided in Supp.Table 15.

REVIEWER 2

Comment 2.1. Sampling strategy is inconsistent throughout the duration of the experiment. This is illustrated in Fig 1B as in six sampling points only two of them are the same. These inconsistencies later translate to results and discussion that are harder to follow.

Response 2.1. We agree with the reviewer that a caveat of our study is the inconsistency of the depths across the duration of the experiment. This was primarily due to our evolving

understanding of the strong stratification of microbial communities across depth which prompted us to increase both the continuity and interval density of the depth profiles as the experiment progressed. In addition, as microbial abundance typically declines sharply with depth, DNA extractions from deep horizons yield little material, which leads to missing data points. We have tried to make this caveat in the design clear from the start of the manuscript, illustrating it in Figure 1B and describing it in the text. Our statistical analysis also takes this into account by intentionally not bundling the approximate depth intervals across years and noting it explicitly in the text (i.e., Figure 3 and Table 1 legends and associated text in Results).

Comment 2.2. We are concerned about the vicinity of the plots, and whether the samples could be considered truly biologically independent at this scale (as shown in Fig. 1A). Moreover, several cores are sampled within the plot which opens a question whether the disturbance could have any effects.

Response 2.2 We thank the reviewer for pointing out the need to clarify the relative positions of the plots and the cores. We have now added the following to the methods:

> "The coordinates corresponding to each of the three paired plots are given in Supplementary Table 1. Plots within a pair are adjacent and located in the same stand conditions to minimize differences in overstory, slope, and soils, with the control plot providing the local reference for the heated one. The distances between the 3 paired experimental plots are 21m, 25m, and 34m. Each plot is circular, 3m in diameter. To minimize the effect of disturbance across different years, soil cores were taken between heating rods and away from temperature probes, maintaining the integrity of the heating system and the monitoring instrumentation, and avoiding repeated sampling in the same plot regions."

Comment 2.3. The authors would like to challenge the glucose-centric model and present a convergence of microbial metabolism on LMW and C1 with increasing depth. However, the experimental set-up and location is very specific and its results cannot serve as a basis of a general model. The experiment has been running in a temperate conifer forest mostly dominated by species from Pinaceae family. Therefore, low soil pH would dictate the composition and functon(including enzymatic activities) of the microbes. Furthermore, the interaction with ectomycorrhizal fungi, abundant in mature forests dominated by species of Pinaceae family, would further drive the selection of other microbial species. Hence, this specific case is not representative of other ecosystems.

Response 2.3. We appreciate the reviewer's thoughtful point regarding the specificity of our experimental site. We acknowledge that these local conditions inevitably shape the specific microbial community composition and their enzymatic activities. However, the functional convergence on low-molecular-weight (LMW) and C1 compounds with increasing depth is a

pattern supported by findings across diverse ecosystems, extending beyond the specific conditions of our site. As we note in the manuscript, the depletion of easily accessible, complex plant inputs with depth generally selects for alternative metabolic strategies. Previous studies have demonstrated that deep soil communities across various ecosystems are consistently enriched in organisms capable of metabolizing C1 and LMW carbon compounds, as well as utilizing inorganic nitrogen forms as energy sources (as cited in Alves et al., 2021). Furthermore, the taxonomic shift we observed with depth, such as the increased relative abundance of Acidobacteriota, Chloroflexota, and Dormibacterota, is a well-documented pattern that is consistent across numerous different ecosystems globally, not just in conifer forests (as cited in Dove et al., 2021).
Our proteogenomic results further align with broad evidence that subsurface microbes persist via atmospheric CO oxidation, alcohol fermentation, and other alternative metabolisms as more favorable substrates and oxygen become limiting with depth (Cordero et al. 2019; Keiluweit et al. 2017; Greening and Grinter 2022). While we do highlight certain site-specific functional capacities in the manuscript, such as the presence of methanol dehydrogenases across 13 phyla in our forest soils, which contrasts with their absence in these phyla within Mediterranean grasslands (Diamond et al. 2019), the overarching metabolic shift towards LMW and C1 cycling represents a generalized adaptation to the resource-limited conditions typical of subsoils. To address your concern, we have updated the discussion section of the revised manuscript to explicitly acknowledge the site-specific characteristics of our temperate conifer forest, while more clearly contextualizing our findings within the broader literature that supports the generalizability of subsoil metabolic convergence on C1 and LMW compounds.

> "Beyond these conifer forest soils, we note that our proteogenomic results align with broad evidence that subsurface microbes persist via atmospheric CO oxidation, alcohol fermentation, and other alternative metabolisms as more favorable substrates and oxygen become limiting with depth (Cordero et al. 2019; Keiluweit et al. 2017; Greening and Grinter 2022)."

Comment 2.4. Figure 3. and Tab. 1 are hard to navigate. This is exacerbated by the inconsistencies in the experimental design.

Response 2.4. This is an effect of the sampling strategy and field realities that we tried to elaborate in our Response 2.1. Figure 3 and Table 1 are already summarized overviews of the full results in Supp Table 15, by year and surface vs. deepest depths, respectively. This choice in their organization was intentional as the short vs. long-term effects should be interpreted in the context of depth intervals, due to the need to consolidate the irregular sampling of depth intervals across years.

Comment 2.5. A strong emphasis is placed on discussion of C and N metabolism; yet, a basic analysis of soil is missing such as C, N, P content and soil pH.

Response 2.5. We appreciate the reviewer's point. The basic soil chemical parameters for these exact experimental plots have been extensively characterized and published in previous studies. Specifically, the slightly acidic soil pH (ranging from 4.5 to 5.5 down the profile) has been previously documented by (Zosso et al. 2021; Ofiti et al. 2021). Similarly, total carbon and nitrogen content, as well as C:N ratios across the soil depth profile, are thoroughly detailed in (Hicks Pries et al. 2017; Soong et al. 2021; Ofiti et al. 2021). To provide readers with this essential context in the revised manuscript, we prominently cited these prior studies in the manuscript to direct them to the existing soil chemical analyses in the discussion (see the Responses 1.1 and 1.2 above).

Comment 2.6. Moreover discussion could expand on how the metabolism of the analysed microbes interconnects with other major organisms present in the soil – plants and fungi.

Response 2.6. We appreciate the reviewer's suggestion and have added material to the discussion to highlight some interconnections:

> "The metabolism of the analyzed soil microbes is tightly coupled to both plants and soil fungi. In surface soils, microbial communities were enriched in traits for metabolizing direct plant inputs, such as plant-derived methanol and pectin from root exudates and litter. However, warming-induced declines in fine root mass (Ofiti et al. 2021) likely exacerbate carbon limitation in the subsoil, restricting plant-derived substrate availability and driving deep-soil microbes toward alternative metabolisms like C1 and CO oxidation. The highly expressed sialidases in deep soils indicate that these bacteria actively scavenge glycoprotein-rich microbial and fungal necromass, unlocking carbon and nitrogen reservoirs possibly generated by fungal turnover."

Comment 2.7. L27 There are studies that have shown the effect of soil depth on microbial communities (e.g. https://doi.org/10.1128/AEM.02673-20). The link with warming effect does not seem smooth and gives the impression being an add-on study.

Response 2.7. We appreciate the reviewer's comment. However, the connection between soil depth and warming is the foundational premise of our study, rather than an add-on. As we explicitly state in the introduction of our manuscript, we "leveraged a long-term whole-soil warming experiment" where the "entire soil profile has been continuously warmed by 4 °C above ambient temperature since 2014". The core hypothesis driving this work is precisely that "depth-stratified microbial traits, in combination with their environmental context... mediate the long-term response of soil respiration to warming". Because this integrated approach is central to our experimental design and is already clearly established in the existing text, we feel the

manuscript naturally links depth and warming, and we have opted not to add any new text to the manuscript.

Comment 2.8. The manuscript contains many unexplained abbreviations both in the text (L55, L58, L65, L84, L85, L91) and in the Tab. 1

Response 2.8. We thank the reviewer for pointing these out. For ANI, NMDS, AOA, AOB, DNRA, and SCFAs we have added the full forms in their first use. For DPANN archaea, we have cited the original reference naming this clade.

REVIEWER 3

Comment 3.1. Line 209 speaks to current earth system models, but cites only papers that are a decade old. There have been advances made in that timeframe to account for soil microbial traits, but still deep soil microbiomes are not considered. The papers cited are excellent, and the point is valid, but perhaps more nuance and more recent citations would help.

Response 3.1. We appreciate the feedback here. We added further nuance on advances towards microbial representation in Earth System Models with more recent citations, yet still stressing that deep soil microbial representation is lacking. Below is the updated relevant text:

> "Even though the importance of microorganisms for global biogeochemical cycles is well recognized, efforts to explicitly incorporate them into Earth System Models have only begun in recent years (Lennon et al. 2024; Berardi et al. 2020; Abramoff et al. 2018; Wieder et al. 2015; Tao et al. 2025). The next generation of ecosystem models is poised to more heavily incorporate trait-based approaches to explicitly model microbial control of organic matter decomposition (Lennon et al. 2024; Berardi et al. 2020; Abramoff et al. 2018; Wan and Crowther 2022), which will necessitate incorporation of microbial traits that are demonstrated to enhance fitness in deeper soils, as well as accounting for their substrates and metabolic/biogeochemical pathways."

Comment 3.2. Figure 1 caption does not adequately indicate the importance of the colors in part B, and connect those colors to the colored soil cores indicated in part A. Along those lines, I think I figured out that the color coding of Figure 1 carries through the part A of Figure 2, which is good but should be given its own legend of some sort, but then part C uses a completely different shading with depth with the tiniest legend I have ever seen. I think part C should also use the shading used in Figure 1 part A, with different color by year and lighter colors with depth, rather than the darker with depth being used now. That consistency would help simplify.

Response 3.2. We agree with the reviewer about these improvements and made small adjustments to Figure 1 and 2. In Figure 1B, in addition to the legend text, we have added an

explicit legend for years and depth and repeated the same legend in Figure 1A. In addition, we expanded the legend text with the following text:

> "Each sampling year is denoted with a distinct color palette, with darker to lighter shades denoting the depth profile from most surface to the deepest."

We also modified the depth to shading mapping in Figure 2C, with bigger legend, to follow the same shading scheme as 1A and 1B (lighter with depth). For analysis across years, we use depth intervals rather than actual depths to accommodate sampling scheme. We therefore used a single color palette for all years so the "binned" depths can be visually compared across depth intervals.

Comment 3.3. Figure 3 – are each of these comparisons indicated with color for significant changes? I do think I like the use of presence/absence of change vs. heat map here, as it is easy to understand.

Response 3.3. The reviewer is correct that color is used to indicate the presence/absence of change for significant changes. This was noted in the legend text.

> "The figure summarizes community-aggregated genome-detected microbial traits (weighted by the coverage of the underlying genomes) **that are significantly different** across control and warming plots (red/blue: increase/decrease with warming)."

Comment 3.4. In the methods, the first sentence of the section "Soil sampling, DNA extraction and metagenome sequencing" was unclear and should be clarified. Also in this section, there is no explanation of the variable depths and variable binning of depth increments in different years, and the approach to the comparisons of data given differences in sampling between years. This justification and explanation is necessary.

Response 3.4. We appreciate the reviewer pointing out these areas for clarification. We have updated the methods section to improve the clarity of the first sentence and explicitly explain our approach to handling the variable depth increments.

First, to clarify the opening sentence, we have revised it to clearly specify the sampling timeline rather than using a broad range: "We collected soil cores from each plot using a 5-cm diameter AMS corer (AMS Inc. Hayward Falls, ID, USA) at six distinct time points: 0.5, 1.5, 2.5, 3.5, 4.5, and 7.5 years after the warming treatment began."

Second, regarding the variable binning of depths, we have expanded upon the phrase "due to field practicalities" to better explain that sampling protocols evolved over the course of this 7.5-year field experiment. However, to ensure these differences did not bias our results, we have clarified our statistical approach in the text. We have explicitly stated that we only compared data using exactly matched depth intervals from the same sampling year.

Specifically, we state in the methods that to test the warming treatment effect, separate linear mixed-effect models were built exclusively for "date and depth matched samples from control and warming plots for which data from all three replicates was available". By restricting our statistical comparisons to perfectly paired depth intervals within the same field season, we avoided any confounding effects caused by differences in depth binning between years. Depth effects were similarly tested using only years where data from all three replicates were available across the entire depth profile.

References

Abramoff, Rose, Xiaofeng Xu, Melannie Hartman, et al. 2018. "The Millennial Model: In Search of Measurable Pools and Transformations for Modeling Soil Carbon in the New Century." *Biogeochemistry* 137 (1-2): 51–71. https://doi.org/10.1007/s10533-017-0409-7.

Allison, Steven D., Matthew D. Wallenstein, and Mark A. Bradford. 2010. "Soil-Carbon Response to Warming Dependent on Microbial Physiology." *Nature Geoscience* 3 (5): 336–340. https://doi.org/10.1038/ngeo846.

Alves, Ricardo J. Eloy, Ileana A. Callejas, Gianna L. Marschmann, et al. 2021. "Kinetic Properties of Microbial Exoenzymes Vary with Soil Depth but Have Similar Temperature Sensitivities through the Soil Profile." *Frontiers in Microbiology* 12 (735282): 735282. https://doi.org/10.3389/fmicb.2021.735282.

Berardi, Danielle, Edward Brzostek, Elena Blanc‐Betes, et al. 2020. "21st‐century Biogeochemical Modeling: Challenges for Century‐based Models and Where Do We Go from Here?" *Global Change Biology. Bioenergy* 12 (10): 774–788. https://doi.org/10.1111/gcbb.12730.

Cordero, Paul R. F., Katherine Bayly, Pok Man Leung, et al. 2019. "Atmospheric Carbon Monoxide Oxidation Is a Widespread Mechanism Supporting Microbial Survival." *The ISME Journal* 13 (11): 2868–2881. https://doi.org/10.1038/s41396-019-0479-8.

Diamond, Spencer, Peter F. Andeer, Zhou Li, et al. 2019. "Mediterranean Grassland Soil C-N Compound Turnover Is Dependent on Rainfall and Depth, and Is Mediated by Genomically Divergent Microorganisms." *Nature Microbiology* 4 (8): 1356–1367. https://doi.org/10.1038/s41564-019-0449-y.

Dove, Nicholas C., Margaret S. Torn, Stephen C. Hart, and Neslihan Taş. 2021. "Metabolic Capabilities Mute Positive Response to Direct and Indirect Impacts of Warming throughout the Soil Profile." *Nature Communications* 12 (1): 2089. https://doi.org/10.1038/s41467-021-22408-5.

Frey, Serita D., Juhwan Lee, Jerry M. Melillo, and Johan Six. 2013. "The Temperature Response of Soil Microbial Efficiency and Its Feedback to Climate." *Nature Climate Change* 3 (4): 395–398. https://doi.org/10.1038/nclimate1796.

Greenfield, Lucy M., Paul W. Hill, Eric Paterson, Elizabeth M. Baggs, and Davey L. Jones. 2018. “Methodological Bias Associated with Soluble Protein Recovery from Soil.” *Scientific Reports* 8 (1): 11186. https://doi.org/10.1038/s41598-018-29559-4.

Greening, Chris, and Rhys Grinter. 2022. “Microbial Oxidation of Atmospheric Trace Gases.” *Nature Reviews. Microbiology* 20 (9): 513–528. https://doi.org/10.1038/s41579-022-00724-x.

Hicks Pries, Caitlin E., C. Castanha, R. C. Porras, and M. S. Torn. 2017. “The Whole-Soil Carbon Flux in Response to Warming.” *Science (New York, N.Y.)* 355 (6332): 1420–1423. https://doi.org/10.1126/science.aal1319.

Keiluweit, Marco, Tom Wanzek, Markus Kleber, Peter Nico, and Scott Fendorf. 2017. “Anaerobic Microsites Have an Unaccounted Role in Soil Carbon Stabilization.” *Nature Communications* 8 (1): 1771. https://doi.org/10.1038/s41467-017-01406-6.

Lennon, J. T., R. Z. Abramoff, S. D. Allison, et al. 2024. “Priorities, Opportunities, and Challenges for Integrating Microorganisms into Earth System Models for Climate Change Prediction.” *mBio* 15 (5): e0045524. https://doi.org/10.1128/mbio.00455-24.

Nicora, Carrie D., Brian J. Anderson, Stephen J. Callister, et al. 2013. “Amino Acid Treatment Enhances Protein Recovery from Sediment and Soils for Metaproteomic Studies.” *Proteomics* 13 (18-19): 2776–2785. https://doi.org/10.1002/pmic.201300003.

Nicora, Carrie D., Kristin E. Burnum-Johnson, Ernesto S. Nakayasu, et al. 2018. “The MPLEx Protocol for Multi-Omic Analyses of Soil Samples.” *Journal of Visualized Experiments: JoVE*, no. 135 (May). https://doi.org/10.3791/57343-v.

Ofiti, Nicholas O. E., Cyrill U. Zosso, Jennifer L. Soong, et al. 2021. “Warming Promotes Loss of Subsoil Carbon through Accelerated Degradation of Plant-Derived Organic Matter.” *Soil Biology & Biochemistry* 156 (108185): 108185. https://doi.org/10.1016/j.soilbio.2021.108185.

Royalty, Taylor M., and Andrew D. Steen. 2019. “Theoretical and Simulation-Based Investigation of the Relationship between Sequencing Effort, Microbial Community Richness, and Diversity in Binning Metagenome-Assembled Genomes.” *mSystems* 4 (5): e00384–19. https://doi.org/10.1128/mSystems.00384-19.

Soong, Jennifer L., Cristina Castanha, Caitlin E. Hicks Pries, et al. 2021. “Five Years of Whole-Soil Warming Led to Loss of Subsoil Carbon Stocks and Increased CO2 Efflux.” *Science Advances* 7 (21): eabd1343. https://doi.org/10.1126/sciadv.abd1343.

Tao, Feng, Yuanyuan Huang, Bruce A. Hungate, et al. 2023. “Microbial Carbon Use Efficiency Promotes Global Soil Carbon Storage.” *Nature* 618 (7967): 981–985. https://doi.org/10.1038/s41586-023-06042-3.

Tao, Jing, William J. Riley, Jinyun Tang, et al. 2025. “Representing Soil Microbial Dynamics and Organo‐mineral Interactions in the E3SM Land Model (ELM‐ReSOM).” *Journal of Advances in Modeling Earth Systems* 17 (10). https://doi.org/10.1029/2024ms004874.

Tian, Ye, Andreas Schindlbacher, Carolina Urbina Malo, et al. 2023. "Long-Term Warming of a Forest Soil Reduces Microbial Biomass and Its Carbon and Nitrogen Use Efficiencies." *Soil Biology & Biochemistry* 184 (109109): Not Available. https://doi.org/10.1016/j.soilbio.2023.109109.

Wan, Joe, and Thomas W. Crowther. 2022. "Uniting the Scales of Microbial Biogeochemistry with Trait‐based Modelling." *Functional Ecology* 36 (6): 1457–1472. https://doi.org/10.1111/1365-2435.14035.

Wieder, W. R., A. S. Grandy, C. M. Kallenbach, P. G. Taylor, and G. B. Bonan. 2015. "Representing Life in the Earth System with Soil Microbial Functional Traits in the MIMICS Model." *Geoscientific Model Development* 8 (6): 1789–1808. https://doi.org/10.5194/gmd-8-1789-2015.

Yu, Wei Hua, Na Li, Dong Shen Tong, Chun Hui Zhou, Chun Xiang (cynthia) Lin, and Chuan Yun Xu. 2013. "Adsorption of Proteins and Nucleic Acids on Clay Minerals and Their Interactions: A Review." *Applied Clay Science* 80-81 (August): 443–452. https://doi.org/10.1016/j.clay.2013.06.003.

Zhang, Qiufang, Jiguang Feng, Xiaojie Li, Yuehmin Chen, Joshua P. Schimel, and Biao Zhu. 2024. "Effect of Field Warming on Soil Microbial Carbon Use efficiency——A Meta-Analysis." *Soil Biology & Biochemistry* 197 (109531): 109531. https://doi.org/10.1016/j.soilbio.2024.109531.

Zosso, Cyrill U., Nicholas O. E. Ofiti, Jennifer L. Soong, et al. 2021. "Whole-Soil Warming Decreases Abundance and Modifies the Community Structure of Microorganisms in the Subsoil but Not in Surface Soil." *SOIL* 7 (2): 477–494. https://doi.org/10.5194/soil-7-477-2021.
