# Supplementary material for: Soil proteogenomics uncover microbial strategies for soil depth-specific carbon cycling under whole-soil warming conditions
Source: Nat Commun. 2026 Oct 13;17:10740. doi: 10.1038/s41467-026-78137-0 (PMC13661901; doi:10.1038/s41467-026-78137-0)
Supplement: Supplementary file 4 [file 41467_2026_78137_MOESM3_ESM.docx]

**Supplementary Data 1. Sample metadata.** Metagenome samples analyzed in this study (n = 95 independent samples).

**Supplementary Data 2. Metagenome metadata and sequencing statistics.** Metagenome metadata, accession ids and statistics (sequencing effort per sample). 101 metagenomes from 95 samples were generated. For samples with multiple metagenomes generated, the corresponding information is separated by commas.

**Supplementary Data 3. Metaproteome raw (EMSL database) and processed (MassIVE database) data accession ids**

**Supplementary Data 4. Metagenome assembly and coassembly statistics.** Assembly statistics for 101 metagenome assemblies and 14 metagenome co-assemblies (115 total assemblies.

**Supplementary Data 5. rpsC sequences and cluster information.** The table lists identified rpsC gene ids (n=12069) across all assemblies, their 99% sequence identity cluster membership, the scaffold ids and their lengths. The gene ids for the centroids are specified in the column “rpsC-gene-id_centroid”.

**Supplementary Data 6. rpsC species groups identified across all metagenome assemblies and their class level lineage.** The table lists the species groups as defined by rpsC clusters (n=3533) detailed in Supp. Table 5 and indexed by the gene-id for the centroid. 3 non-prokaryotic clusters were removed (rpsC_lf.cluster3429, rpsC_lf.cluster1061, rpsC_lf.cluster1736).

**Supplementary Data 7. Species group coverage of reads from each sample normalized by sequencing yield per sample.** For each species group, the normalized per base pair read coverage for the longest scaffold representing the species group cluster across samples. Normalized coverage for each species group from a sample was calculated as follows: (mean coverage of the scaffold/sequencing depth in bp*10E8)

**Supplementary Data 8. All dereplicated bins with completeness > 50, contamination < 25 and their mean coverage profiles across samples.** For each genome bin, the table lists the source metagenome assembly, GTDB taxonomic classification, completeness and contamination metrics, size and N50 length, followed by mean coverage across all samples.

**Supplementary Data 9. Genome bins associated with rpsC clusters.** Table linking rpsC clusters to MAGs for analysis of bin representativeness of the communities (see Supp. Figures 6 and 7)

**Supplementary Data 10. Permutational multivariate analysis of variance partitioning of weighted UniFrac distances for the microbial communities.** Microbial community structure was determined based on the rpsC based species clusters. The table is for permutational multivariate analysis of variance table from adonis2 using weighted phylogenetic distance (weighted UniFrac distance)  with the model “weighted_UniFracdist ~ depth * treatment”.

**Supplementary Data 11. Warming treatment and depth effect on MAG abundance - Linear mixed effect modeling results for testing for treatment and depth effects separately using normalized coverage.**

**Supplementary Data 12. Profiled traits through HMMs and HMM information (name, source, description, and KEGG cross reference when relevant).**

**Supplementary Data 13. Trait profiling of MAGs (presence/absence (1/0) or counts)**

**Supplementary Data 14. Community aggregated trait abundances based on MAG traits and MAG coverages.**

**Supplementary Data 15. Warming treatment and depth effect on community weighted traits.**

**Supplementary Data 16. Spectral counts for the proteins predicted from metagenomes**

**Supplementary Data 17. Normalized spectral abundance factors (NSAF) for the proteins predicted from metagenomes**

**Supplementary Data 18. Total Normalized spectral abundance factors (NSAF) assigned to annotation categories**

**Supplementary Data 19. Detection of protein families across replicate plots.**
